# Gelingens- und Hindernisfaktoren bei der Implementierung von Gesundheitsförderung in Kitas und Grundschulen anhand von 4 ausgewählten Projekten

**DOI:** 10.1007/s00103-024-03935-0

**Published:** 2024-08-20

**Authors:** Anna Scheffler, Laura Klocker, Angelika Puls, Eva Hummers, Iris Demmer

**Affiliations:** 1 Institut für Allgemeinmedizin, Universitätsmedizin Göttingen, Göttingen, Deutschland; 2 Gesundheitsregion Göttingen/Südniedersachsen, Göttingen, Deutschland; 3 Gesundheitsamt für Stadt und Landkreis Göttingen, Göttingen, Deutschland

**Keywords:** Primärprävention, Kindergesundheit, Implementierungsmodell, CFIR, Präventionsforschung, Primary prevention, Child health, Implementation model, CFIR, Prevention research

## Abstract

**Hintergrund:**

Gesundheitsförderung (GF) ist eine komplexe, politisch geforderte Aufgabe. Die Implementierungsgüte korreliert mit der Wirksamkeit von GF. Sie erfolgreich zu implementieren, erfordert die Beachtung von Kontextbedingungen, was in Wirksamkeitsstudien eher unterbleibt. Ziel ist es, Gelingens- und Hindernisfaktoren für die Implementierung von GF in Kindertagesstätten und Grundschulen anhand von 4 Projekten (*Fit fürs Leben, fit für pisa* *+*, Buchprojekt *Sonnige Traurigtage* und *The Daily Mile)* in der Gesundheitsregion Göttingen/Südniedersachsen zu identifizieren. Die Auswirkungen der COVID-19-Pandemie werden als implementierungsrelevant für diese Projekte angenommen.

**Methoden:**

In 24 semistrukturierten Interviews und 2 Fokusgruppendiskussionen wurden im Zeitraum 08/2021–03/2022 hauptsächlich Lehrer*innen und Erzieher*innen zur Umsetzung der Projekte in ihren Tätigkeitsbereichen befragt. Nach Transkription wurde mittels qualitativer Inhaltsanalyse ausgewertet. Das *Consolidated Framework for Implementation Research* unterstützte die Analyse.

**Ergebnisse:**

Insgesamt wurden 22 Faktorengruppen identifiziert, auf deren Basis 22 Handlungsempfehlungen für die Implementierung von GF formuliert wurden. Im Vordergrund standen Gelingensfaktoren auf der Individuen- und Interventionsebene. Die COVID-19-Pandemie wirkte sich positiv auf den Stellenwert der GF aus, obgleich ihre Umsetzbarkeit erschwert war.

**Diskussion:**

Zahlreiche Ergebnisse zur Implementierung von GF in kindlichen Lebenswelten konnten durch die Studie bestätigt werden. Auffällig ist der hohe Stellenwert der Individuenfaktoren, was sich auf die Befragung projektnaher Interviewpersonen zurückführen lassen könnte. Die Studienergebnisse tragen zur Weiterentwicklung von Implementierungsstrategien in der GF bei.

**Zusatzmaterial online:**

Zusätzliche Informationen sind in der Online-Version dieses Artikels (10.1007/s00103-024-03935-0) enthalten.

## Abstract (en)

**Background:**

Health promotion (HP) is a complex, politically required task. The quality of implementation correlates with the effectiveness of health promotion initiatives. Successful implementation requires consideration of contextual conditions, which tend to be neglected in effectiveness studies. The aim is to identify success factors and obstacles to the implementation of HP in daycare centers and elementary schools based on 4 projects (*Fit fürs Leben*, *fit für pisa* +, Buchprojekt *Sonnige Traurigtage*, and The Daily Mile) in the Göttingen/South Lower Saxony region. The effects of the COVID-19 pandemic are assumed to be relevant for the implementation of these projects.

**Methods:**

In 24 semi-structured interviews and 2 focus group discussions between August 2021 and March 2022, practitioners were asked about the implementation of the four projects in their areas of activity. After transcription, the interviews were analyzed using qualitative content analysis. The Consolidated Framework for Implementation Research supported the analysis.

**Results:**

A total of 22 groups of factors were identified, on the basis of which 22 recommendations for action for GF implementation were formulated. Success factors at the individual and intervention level were at the forefront. The COVID-19 pandemic had a positive impact on the importance of HP, even though its feasibility was hampered.

**Discussion:**

Numerous findings on the implementation of HP in children’s living environments were confirmed by the study. The high significance of the individual factors is striking, which could be due to the survey of interviewees being familiar with the projects. The study results contribute to the further development of implementation strategies in HP.

## Hintergrund

Gesundheitsförderung (GF) wird in Deutschland politisch für zahlreiche Lebenswelten (LW) gefordert [1, 2]. Kindliche LW wie Kindertagesstätten (Kitas) und Grundschulen nehmen einen besonderen Stellenwert bei der Umsetzung von Maßnahmen zur GF ein [3, 4].

Anders als bei medizinischen Interventionen ist der Wirksamkeitsnachweis bei komplexen Public-Health-Maßnahmen erschwert [5], da sie in Wechselwirkung mit ihrem Umsetzungskontext stehen. Es zeigte sich, dass die Implementierungsgüte die Effektivität begünstigt [6]. Zwar überwiegen in der Präventionsforschung gegenwärtig Fragen zur Wirksamkeit jene zur Implementierung, dennoch sollten beide Interessenpunkte verfolgt werden [7]. Bei Praktiker*innen stehen Überlegungen zur Machbarkeit im Vordergrund [8].

Insgesamt wächst das Interesse an der Implementierungsforschung. Es ist bedeutsam zu verstehen, wie GF umgesetzt werden kann, um die beabsichtigten Wirkungen zu erzielen [6, 9]. Ein umfassendes Verständnis für Implementierungsprozesse bei komplexen Interventionen trägt dazu bei, ihre Effektivität zu erhöhen [6, 10, 11]. Wie idealerweise evidenzbasierte Maßnahmen erfolgreich in realen Settings umgesetzt werden können, liegt im Fokus der Implementierungswissenschaften [12, 13]. Wirkungsvolle Maßnahmen in der GF basieren sowohl auf praktischen als auch auf wissenschaftlichen Erkenntnissen [14, 15]. Ein umfassendes Wissen zur Implementierung von GF kann dazu beitragen, dass Interventionen in unterschiedlichen Settings effektiver umgesetzt und disseminiert werden können [16]. Gegenwärtig unterbleibt noch häufig die Verstetigung und Dissemination wirksamer Projekte [17].

Für die Implementierung von komplexen Interventionen wurden zahlreiche Rahmenmodelle entwickelt, die Implementierungsaspekte in Kategorien abbilden [18]. Darunter hat sich das *Consolidated Framework for Implementation Research* (CFIR) nach Damschroder et al. [19] als nützliches Determinanten-Framework für die Analyse implementierungsrelevanter Faktoren erwiesen [18, 20]. Das CFIR umfasst 5 Ebenen: Die Implementierung einer Intervention ist von ihren Interventionsmerkmalen (1. Intervention) bestimmt und wird im Zusammenhang mit ihrem umgebenden Kontext (2. Inner Setting) und den dort interagierenden Individuen (3. Individuen) betrachtet. Zusätzlich beeinflusst das äußere Umfeld (4. Outer Setting) mit seinen gesellschaftlichen, kulturellen und politischen Eigenschaften den gesamten Implementierungsprozess (5. Prozess).

Die COVID-19-Pandemie hat nicht nur auf die Gesundheit von Kindern eingewirkt [21, 22], sondern auch auf die Durchführung von Maßnahmen, die Kinder im Aufbau von Gesundheitsressourcen bestärken sollten [23]. Dieser Aspekt ist aufgrund seiner Aktualität noch nicht hinreichend erforscht. Kürzlich ergänzten Damschroder et al. in ihrer überarbeiteten Version die Outer-Setting-Domäne um das Konstrukt „kritische Ereignisse“ *(Critical Incidents)* und benannten als Beispiel die COVID-19-Pandemie [24], was deren Einfluss auf Implementierungsprozesse hervorhebt.

Im Jahr 2010 wurden erstmals „Gesundheitsregionen“ als Modellversuch zur Etablierung kommunaler Gesundheitsnetzwerke und -strukturen in Niedersachsen initiiert und 2014 im Bundesland ausgeweitet [25]. Sie hatten eine koordinierte GF und Gesundheitsversorgung zum Ziel [25]. In der Gesundheitsregion Göttingen/Südniedersachsen fanden in diesem Zusammenhang u. a. 4 Präventionsprojekte an Kitas und Schulen statt: *Fit fürs Leben, fit für pisa* *+*, Buchprojekt *Sonnige Traurigtage* und *The Daily Mile*.

Vor dem Hintergrund, dass sich die Implementierungsgüte auf die Effektivität einer gesundheitsfördernden Intervention auswirkt, wird mit der vorliegenden Arbeit das Ziel verfolgt, Gelingens- und Hindernisfaktoren, die die Präventionsarbeit in Kitas und Schulen beeinflussen, anhand der Erfahrungen von Praxisexperten*innen mit der Umsetzung der 4 Projekte in der Gesundheitsregion Göttingen/Südniedersachsen zu identifizieren. Es wird angenommen, dass diese Faktoren auf vergleichbare Kontexte übertragbar sind. Die Ergebnisse sollen einerseits einen Beitrag für die Implementierungs- und Präventionsforschung leisten und andererseits den Praktiker*innen Anregungen in Form von Handlungsempfehlungen bieten. Im Untersuchungszeitraum war die COVID-19-Pandemie gegenwärtig, deren Auswirkungen als implementierungsrelevant angenommen und in der Analyse berücksichtigt wurden.

## Methoden

Es handelt sich um eine qualitative Forschungsarbeit, in der 39 Studienteilnehmende in 24 leitfadengestützten Einzelinterviews und 2 Fokusgruppen im Zeitraum August 2021 bis März 2022 einmalig befragt wurden. Das Sampling erfolgte kriterienbasiert. Es wurden nur Fachpersonen ausgewählt, die sich im Erhebungszeitraum aktiv an der Umsetzung der 4 ausgewählten Projekte (Tab. 1) in der Gesundheitsregion Göttingen/Südniedersachsen beteiligten. Potenzielle Studienteilnehmende konnten identifiziert werden, indem Einrichtungsleitungen und Projektanbietende kontaktiert wurden.

**Tab. 1:** Steckbriefe der 4 ausgewählten Präventionsprojekte in der Gesundheitsregion Göttingen/Südniedersachsen

Merkmale	*Fit fürs Leben*	*Fit für pisa* *+*	Buchprojekt *Sonnige Traurigtage*	*The Daily Mile*
Umsetzungsort	Stadt und Landkreis Göttingen	Stadt und Landkreis Göttingen	Landkreis Northeim	Landkreis Northeim (internationale Initiative)
Beginn in der Gesundheitsregion Göttingen/Südniedersachsen	Juli 2019	August 2012	März 2021	Oktober 2019
Thematische Ausrichtung	Bewegung	Bewegung	Psychosoziale Gesundheit Stärkung von Kindern psychisch kranker Eltern	Bewegung
	Ernährung	Ernährung		
	Chancengleichheit beim Schulstart	Gesundheitskompetenz		
Ansatz	Universell	Universell	Selektiv/indiziert	Universell
Strategie	Verhaltensprävention	Verhaltensprävention	Verhaltensprävention	Verhaltensprävention
Setting	Kitas	Grundschulen	Grund- und Förderschulen	Grundschulen
Initiatoren	Öffentlicher Gesundheitsdienst Göttingen	Sportclub ASC Göttingen, Kassenärztliche Vereinigung Niedersachsen, Universitätsmedizin Göttingen	Arbeitsgruppe unterschiedlicher Beratungsstellen aus dem Landkreis Northeim, u. a. Kinderschutzbund und Bündnis gegen Depression	Gesundheitsregion Göttingen/Südniedersachsen

Die 4 Projekte wurden in Kooperation mit Mitarbeitenden der Gesundheitsregion Göttingen/Südniedersachsen ausgewählt. Die Projekte sind von Forschungsinteresse, da sie eine große Anzahl von Kindern in der Region erreichen, eine gewisse Umsetzungsdauer aufweisen (Projektbeginn, Tab. 1) und durch ein politisches Engagement unterstützt werden, was ihren bisherigen Implementierungserfolg verdeutlicht. *Fit fürs Leben* bezweckt Chancengleichheit beim Schulstart durch eine frühe Prävention. *Fit für pisa* *+ *soll mithilfe eines Patenarztmoduls sowie Ernährungs- und Bewegungsmodulen Übergewicht und Adipositas im Kindesalter vorbeugen. *Sonnige Traurigtage* bietet Lehrenden und Kindern eine Hilfestellung bei kindlichen Notlagen infolge einer psychischen Erkrankung ihrer Eltern. Es soll die psychische Belastung von Kindern mindern, indem es sie informiert und ihnen Hilfestellen aufzeigt. Das internationale Projekt *The Daily Mile* zielt mit täglichen Bewegungseinheiten auf die Vermeidung von Übergewicht und die Vermittlung von Bewegungsfreude bei Kindern ab, es liegen bereits umfassende Studienergebnisse diesbezüglich vor [26].

**Abb. 1:**
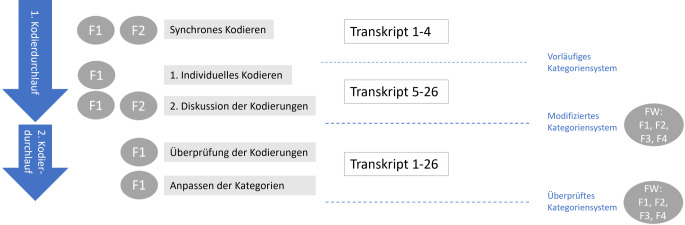
Ablauf des konsensuellen Kodierens und der Erstellung des Kategoriensystems (eigene Abbildung). *F* Forscher*in, *FW* Reflexion in der Forschungswerkstatt

20 von 24 Einzelinterviews wurden im persönlichen Kontakt in den Kitas und Grundschulen durchgeführt, die übrigen Interviews sowie die beiden Fokusgruppen fanden online statt. Der Interviewleitfaden sowie die Fokusgruppen-Inputs sind im Onlinematerial 1 zu diesem Artikel einsehbar. Für die Befragung der Experten*innen für das Projekt *fit für pisa* *+ *wurde das Format von Fokusgruppeninterviews gewählt, da die Projektarbeit durch die Kooperation einzelner Berufsgruppen geprägt war. Die durchschnittliche Länge der Interviews betrug 26 min, die Fokusgruppen dauerten 51 min und 58 min. Die Audiodaten wurden von der Erstautorin wörtlich transkribiert und anschließend, unterstützt durch die Analysesoftware MAXQDA (Versionen 2020 und 2022; VERBI GmbH, Berlin, Deutschland) [27, 28], mittels qualitativer Inhaltsanalyse (QIA) ausgewertet. Das Verfahren ist angelehnt an die inhaltlich strukturierende Inhaltsanalyse nach Kuckartz [29]. Das transkribierte Interviewmaterial wurde in 2 Durchgängen vorwiegend induktiv kodiert. Der konsensuelle Kodierprozess wird grafisch in Anlehnung an Becker et al. [30] in Abb. 1 dargestellt.

Als Interpretationsrahmen für die Analyse von Gelingens- und Hindernisfaktoren wurde das CFIR ausgewählt [19], um sie aus einer Mehrebenenperspektive zu erfassen.

## Ergebnisse

Die Ergebnisse dieser Arbeit basieren auf der Datenerhebung mit 39 Interviewpersonen, deren Charakteristika in Tab. 2 gezeigt sind. Das Kategoriensystem mit 13 Haupt- und 52 Unterkategorien, das sich aus der QIA ergab, ist im Onlinematerial 2 zu finden. Es baut auf 1600 Kodierungen auf und stellt die Basis der CFIR-unterstützten Datenanalyse dar. In den nachfolgenden Abschnitten wird der Begriff „Projekt“ in Analogie zur Terminologie des CFIR von dem Begriff „Intervention“ abgelöst.

**Tab. 2:** Beschreibung der Stichprobe

Merkmale	*Fit fürs Leben* (*n* = *9*)	*Fit für pisa* + (*n* = *16*)	Buchprojekt *Sonnige Traurigtage* (*n* = *7*)	*The Daily Mile *(*n* = 7)	Gesamt (*N* = 39)
*Alter (Jahre)*					
Mittelwert	48,00	48,33	55,00	40,86	46,71
Altersspanne	30–64	25–78	32–63	29–60	25–78
*Beruf und Tätigkeitsfeld*					
Lehrer*in (davon mit Leitungsfunktion)	0 (0)	5 (3)	3 (0)	7 (2)	15 (5)
Erzieher*in (davon mit Leitungsfunktion)	6 (1)	0 (0)	0 (0)	0 (0)	6 (1)
Pädagogische Fachpersonen (u. a. Übungsleiter*innen)	3	3	0	0	6
Schulsozialarbeiter*in	0	0	4	0	4
Ärzt*in	0	4	0	0	4
Projektkoordinator*in	0	4	0	0	4
*Geschlecht*					
Weiblich	8	9	6	4	27
Männlich	1	7	1	3	12
Divers	0	0	0	0	0
*Einrichtung*					
Kita	6	0	0	0	6
Schule	0	8	7	7	22
Keine feste Einrichtungszugehörigkeit	3	8	0	0	11

### Implementierungsrelevante Faktoren

**Abb. 2:**
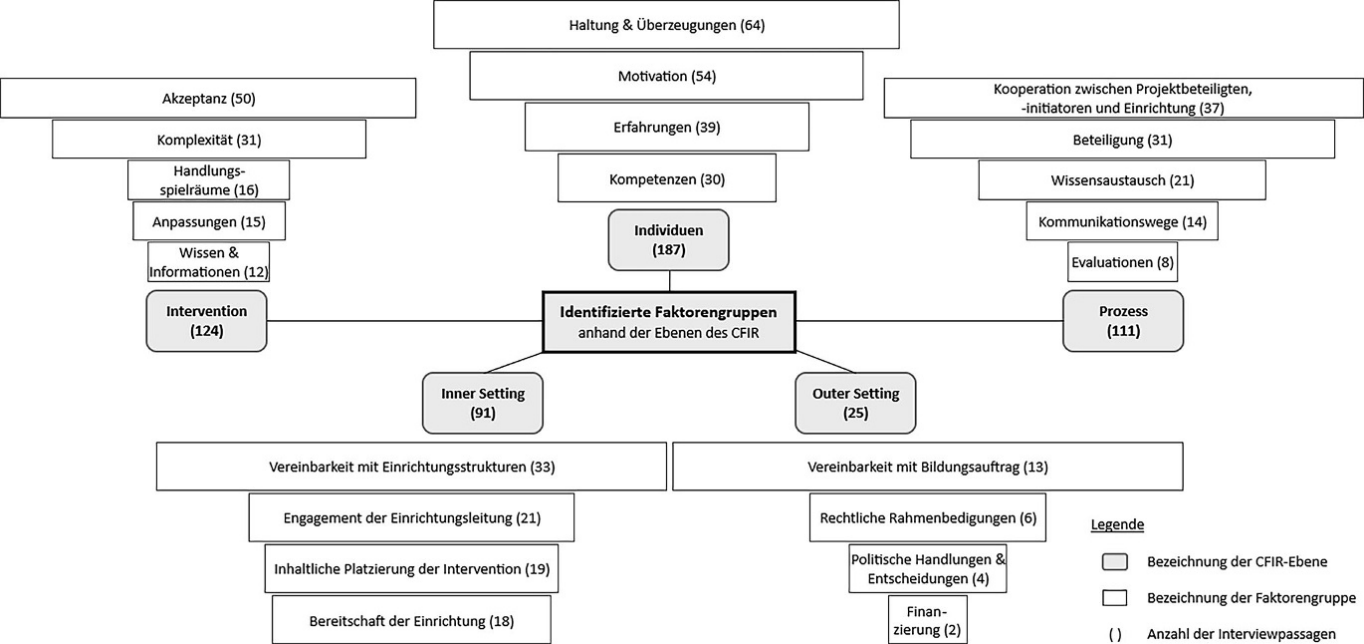
Identifizierte Faktorengruppen anhand der Ebenen des CFIR (Consolidated Framework for Implementation Research [19]; eigene Abbildung)

Insgesamt wurden 538 Interviewpassagen zu 22 Faktorengruppen zusammengefasst und den Ebenen des CFIR (*Individuen, Intervention, Prozess, Inner Setting* und *Outer Setting*) zugeordnet (Abb. 2). Im Folgenden werden die implementierungsrelevanten Faktoren (Gelingens- und Hindernisfaktoren) dargestellt und anhand einzelner Beispielzitate (Tab. 3) illustriert. Als Quintessenz werden datenbasiert Handlungsempfehlungen abgeleitet.

**Tab. 3:** Illustrierende Beispielzitate

Nr.	Interviewzitat (Berufs‑/Tätigkeitsfeld, Projektname)
Z1	„Also was mich noch antreibt, ist einfach die Idee primäre Prävention zu machen und das ist eigentlich was relativ Einfaches. Und das wird in unserer Gesellschaft einfach viel zu wenig gelebt“ (Ärzt*in, *fit für pisa* *+*)
Z2	„... habe Biologie und Kunst studiert. Und naja bei Biologie kommt auch die Gesundheitserziehung dran. … Gesundheitserziehung ist mir schon eine Sache, die mir durchaus am Herzen liegt“ (Lehrer*in, *Sonnige Traurigtage*)
Z3	„Und von daher habe ich hier mit dem Buch noch nicht gearbeitet, weil ich nicht etwas aufbrechen wollte, was dann in der Familie vielleicht so nicht benannt wird“ (Sozialarbeiter*in, *Sonnige Traurigtage*)
Z4	„Also ich glaube, dass eigentlich niemand Einwände hätte, wenn ich so darüber nachdenke. Es sei denn, es ist jetzt jemand da, der selber irgendwie psychisch krank ist, dem das irgendwie unangenehm ist“ (Lehrer*in, *Sonnige Traurigtage*)
Z5	„Da ich schon über dreißig Jahre in diesem Beruf tätig bin, habe ich da schon so einiges, was ich auch an Fortbildungen und an Erfahrungen gesammelt habe, was ich dann auch miteinbringen konnte, denke ich ja. So kann man es sagen“ (Erzieher*in, *Fit fürs Leben*)
Z6	„Und auch die Reduzierung, solche Geschichten waren halt auch sowas, wo wir dann halt gesagt haben, weil wir irgendwann gemerkt haben, fünf Tage die Woche ist vielleicht für die Kinder ein bisschen demotivierend irgendwann“ (Lehrer*in, *The Daily Mile*)
Z7	„Die Eltern haben sich bedankt und fanden ganz interessant und interessant, was hier gemacht wird. Und haben gesagt, dass es toll ist, dass das angeboten wird. Aber jetzt so viel mehr nicht“ (Erzieher*in, *Fit fürs Leben*)
Z8	„Also wir machen ja auch immer mal so eine Flitzepause. Eine kurze Pause, wo die einfach fünf Runden über den Schulhof. Das ist die abgespeckte Variante sozusagen, die wir beibehalten haben, dass sie das zumindest machen“ (Lehrer*in, *The Daily Mile*)
Z9	„… es gibt tatsächlich auch Kollegen, die sich ganz sicher fühlen in ihrer Struktur und in ihrem Lehrplan. Und das haben sie geplant und das ziehen sie durch[.] Und sie haben Angst, dass wenn da neue Leute reinkommen, sie die Klasse vielleicht durcheinanderwirbeln. Man weiß nicht, wie es läuft“ (Lehrer*in, *fit für pisa* *+*)
Z10	„… Damit auch Eltern es besser wahrnehmen. Also ich würde es jetzt so sagen, Eltern nehmen es bei uns nur punktuell wahr, wenn wieder irgendetwas an Aktionen war“ (Erzieher*in, *Fit fürs Leben*)
Z11	„Und wenn jemand es außer uns macht, bringt es für uns auch Freiraum … Weil wenn wir unseren täglichen Alltag haben, ist so viel, was wir machen müssen. Auch von der Bürokratie. Wenn jemand hier ist, es mit den Kindern durchführt, entlastet es und wenn man dabei sein kann, kann man Kinder beobachten“ (Erzieher*in, *Fit fürs Leben*)
Z12	„… das ist eine Frage des Wollens. Also wenn man das will, denke ich mal, dann kriegt man das auch hin. Und dann ist man möglicherweise an der ein oder anderen Stelle vielleicht auch ein bisschen flexibler, das wird für alle so sein, also sowohl für die Schule als auch für die Rahmenbedingungen, die das Projekt so will“ (Lehrer*in, *fit für pisa* *+)*
Z13	„Also in den Kerncurricula taucht diese Thematik an überhaupt gar keiner Stelle auf. Ich müsste hier jetzt schon arg vom Gleis abweichen und andere Sachen krachen lassen und dafür Platz einräumen. Und den habe ich einfach nicht. Schon gar nicht bei vierzig Wochenstunden. Ist einfach so. Nein, also ich habe keine Möglichkeiten, Spielraum auch nicht“ (Lehrer*in, *Sonnige Traurigtage*)
Z14	„Und es gefällt uns eigentlich sehr gut, weil es ja einmal diesen diese zusätzliche Bewegung ähm beinhaltet und einmal aber auch ähm gesunde Ernährung mitbeinhaltet. Und das passt einfach wie Faust aufs Auge in unser Schulprogramm“ (Lehrer*in, *fit für pisa* *+*)
Z15	„Wir können ja jetzt nicht sagen, wir machen da jetzt nur noch Sport und Bewegung. Wir haben da leider auch ganz viele andere Dinge, die wir hier zu tun haben, die vorrangig sind“ (Lehrer*in, *The Daily Mile*)
Z16	„Ja, es war schon aufwändig für die [Projektorganisation], das zu koordinieren, weil die Kindergärten in diesen letzten anderthalb Jahren so viel Stress hatten mit den Corona-Auflagen und teilweise nicht raus durften. Und dies nicht durfte und das nicht durften“ (pädagogische Fachperson, *Fit fürs Leben*)
Z17	„Weil gerade jetzt während Corona hat man ja oft auch gehört, dass der Bewegungsmangel quasi da ist, dass sich weniger bewegt wird, weil eben mehr zuhause stattfindet. Keiner ähm keiner keine Vereinssportarten mehr möglich sind und und und. Und da haben wir als Schule, wie gesagt unter Einhaltung der ganzen Corona-Bedingungen, eben die Möglichkeit, da wenigstens ein wenig sportliche Aktivität mit reinzubringen“ (Lehrer*in, *The Daily Mile*)
Z18	„Ja wir hatten ja teilweise geschlossen. Oder waren hier total eingeschlossen in unseren Gruppen, weil wir gar nicht mischen durften und so weiter. Und da war auch nicht Fit fürs Leben primär im Vordergrund“ (Erzieher*in, *Fit fürs Leben*)

#### Individuenebene

Eine positive offene Grundeinstellung als Faktor in der Gruppe *Haltung und Überzeugungen* unterstützt die Implementierung und Aufrechterhaltung von Interventionen. Eine zuträgliche Haltung zeigte sich in einem vorhandenen Problembewusstsein (Z1), in der Bereitschaft, eigene zeitliche Ressourcen einzusetzen, und in der Bedeutung, die der Intervention beigemessen wurde. Vereinzelt wurde GF auch als mit der Berufstätigkeit einhergehende Aufgabe betrachtet (Z2). Die Aussicht, mit der Intervention Positives für die Zielgruppe zu bewirken, unterstützte das Engagement.

Die Faktorengruppe *Motivation *beinhaltet überwiegend intrinsische Motivationsgründe, die häufig mit dem beruflichen Selbstverständnis verbunden waren. In wenigen Fällen bestanden Bedenken gegenüber Interventionen. Zum einen wurden negative Auswirkungen auf die Zielgruppe (Z3) und zum anderen auf die Fachpersonen vermutet, wie z. B. Überschreiten der Belastbarkeit (Z4).

Positive *Erfahrungen* mit Interventionen begünstigten, dass die Motivation von Individuen aufrechterhalten und die Umsetzung verstetigt wurde. Die Beteiligung an neuen Angeboten wurde durch vorhandene, positive Erfahrungen mit Projekten zur GF insgesamt unterstützt.

*Kompetenzen* beziehen sich darauf, inwiefern Vorwissen, Fähig- und Fertigkeiten bei der Interventionsdurchführung eingesetzt werden können. Vielfach sahen sich Individuen aufgrund ihrer Kenntnisse, die mit ihrer Berufsausbildung oder -tätigkeit erworben wurden, zur Durchführung von GF befähigt (Z5), wodurch zum Teil auf eine gezielte Vorbereitung der Fachpersonen verzichtet werden konnte. Einige Befragte informierten sich eigenständig über die Intervention, um ihre Kompetenzen für diese Aufgabe zu erweitern.

#### Interventionsebene

Auf die *Akzeptanz* einer Intervention wurde geschlossen, wenn sie als kindgerecht, altersentsprechend oder inhaltlich geeignet bewertet wurde. Nicht mit dem Setting und der Zielgruppe kompatible Interventionen wurden als „nicht akzeptabel“ interpretiert. Bei ausgeprägter Sinnhaftigkeit, z. B. bei hohem Bedarf, erhöhte sich die Akzeptanz einer Intervention. Teilweise wurden Interventionen adaptiert, um die Akzeptanz der Zielgruppe aufrechtzuerhalten (Z6). Selten wurde thematisiert, inwiefern Eltern die Projektziele und -inhalte kannten oder akzeptierten. Insgesamt nahmen Eltern eine passive Rolle ein (Z7).

Die *Komplexität* einer Intervention bezieht sich auf die Schwierigkeit, sie in der LW umzusetzen. Eine geringe Komplexität wurde darauf zurückgeführt, dass Interventionen ohne Vorbereitungen und mit geringem Organisationaufwand umsetzbar waren oder als einfach bewertet wurden. Auf eine hohe Komplexität wiesen Unklarheiten und Unsicherheiten, wie z. B. Gedanken zu unerwünschten Auswirkungen, hin (Z3). Besonders bei hoher Komplexität scheint es bedeutsam zu sein, dass Projektinitiatoren Inhalte gegenüber den pädagogischen Fachpersonen vermitteln.

Wenn Interventionen *Handlungsspielräume* bieten, können sie individuell an die Eigenschaften, Interessen und Fähigkeiten der Kinder sowie die Gegebenheiten vor Ort angepasst werden. Flexibles Handeln beinhaltete auch den Einsatz eigener Fähigkeiten und eigenständiges Finden von Lösungen, was z. B. bei wechselnden Wetterbedingungen oder Materialien notwendig war.

Neben individuellen Entscheidungen wurden auch *Anpassungen* durch mehrere Individuen innerhalb einer Einrichtung vorgenommen. Diese Adaptierungen bezogen sich vor allem auf die Häufigkeit und Dauer von Interventionen infolge pandemiebedingter Einschränkungen. Nicht selten ermöglichten erst Anpassungen die Umsetzung, was sie eher als Voraussetzung denn als Gelingensfaktor klassifiziert (Z8). Interventionen flexibel in Abläufe einzuflechten und auf die vorhandenen Ressourcen abzustimmen, waren wichtige Umsetzungsstrategien.

*Wissen und Informationen* über Interventionen befähigen die Beteiligten zur aktiven Mitarbeit, was eine wichtige Voraussetzung für Implementierungstreue ist. Obwohl vielfach von vorhandenen schriftlichen Informationen berichtet wurde, zeigten sich auch Informationsdefizite, die fehlende Kenntnisse über die Zielsetzung oder Ansprechpersonen einer Intervention betrafen. Im Fall des Buchprojekts zeigte sich, dass die Intervention aufgrund von fehlender Information gar nicht als solche wahrgenommen wurde und erst die Studienteilnahme zur Auseinandersetzung mit *Sonnige Traurigtage* in der LW Schule anregte.

#### Prozessebene

Häufig war eine *Kooperation zwischen Projektbeteiligten, -initiatoren und Einrichtung* vorgesehen, ohne dass auf vorbestehende Arbeitsbeziehungen zurückgegriffen werden konnte, was eine koordinierte Aufgabenzuweisung und -verteilung erforderte. Idealerweise begegneten sich alle Akteure*innen auf Augenhöhe und mit gegenseitiger Wertschätzung ihrer persönlichen und fachlichen Kompetenzen. Anhaltendes Engagement von Projektinitiatoren wurde gelobt. Auch die Berücksichtigung von Einrichtungswünschen war bedeutsam. Bei 2 Projekten wurde die Sorge vor einer Bevormundung und die Verunsicherung in der eigenen Struktur (Z9) durch einrichtungsfremde Projektbeteiligte geäußert. Dies verdeutlicht, wie wichtig es ist, zwischenmenschliche Interaktionen in Implementierungsprozessen zu berücksichtigen.

*Beteiligung *nimmt einen besonderen Stellenwert ein. Einige Praxisexperten*innen wünschten sich, stärker in die Projektgestaltung eingebunden zu werden. Andere Stimmen lobten, dass eigene Ideen zur Interventionsumsetzung aktiv eingebracht werden konnten. Darüber hinaus wurde gefordert, Eltern stärker miteinzubeziehen (Z10). Es erscheint insgesamt wichtig, das Maß der Beteiligung mit dem Wunsch der Individuen, einbezogen zu werden, abzustimmen. Einzelne schätzten beispielsweise das Einnehmen einer passiven Rolle, da diese mit Entlastung durch externe Projektbeteiligte oder der Gelegenheit, Kinder beobachten zu können, einherging (Z11). Insgesamt zeigte sich eine stärkere Beteiligung von Einrichtungsleitungen als von -mitarbeitenden.

Für den *Wissensaustausch* eigneten sich Treffen in Einrichtungen, bei denen Informationen und Vorschläge zur Umsetzung unterbreitet wurden. Daneben fand ein informeller Austausch statt, z. B. in Form eines kollegialen Austauschs mit anderen Institutionen. Außerdem wurde der Zugang zu schriftlichen Wissensquellen von den Fachpersonen wertgeschätzt.

Eine gelingende Zusammenarbeit erforderte, dass geeignete *Kommunikationswege* vorhanden waren und genutzt wurden. Mehrmals wurde die mündliche gegenüber der schriftlichen Kommunikation vorgezogen – Dienstbesprechungen wurden häufig genutzt. Bei der Kommunikation zwischen einrichtungsfremden Beteiligten und der Einrichtung wurde häufig der Weg über die Leitung gewählt, was ihre wichtige Funktion unterstreicht.

Die *Evaluation* fokussierte zum einen die Umsetzung von Interventionen. Zum anderen war es für die Befragten wichtig, Interventionsinhalte gemeinsam auf die individuellen Bedürfnisse von Kindern sowie Mitarbeitenden einer Einrichtung abzustimmen. Dieser Faktor konnte zur Akzeptanz einer Intervention beitragen. Häufig wurden Interventionen informell evaluiert, indem ihre Machbarkeit in Besprechungen reflektiert wurde.

#### Inner-Setting-Ebene

Die *Vereinbarkeit mit Einrichtungsstrukturen *war eher eine Voraussetzung als ein Gelingensfaktor. Die Haltung der Individuen bestimmte darüber, wie Ressourcen und Strukturen zugunsten eines Projekts eingesetzt und ggf. angepasst wurden (Z12). Neben zeitlichen und personellen Ressourcen waren für die Bewegungsförderung auch Räumlichkeiten, wie z. B. Sporthallen oder -plätze, notwendig.

Der Einrichtungsleitung obliegt eine Schlüsselfunktion, da sie in der Regel über die Teilnahme an einer Intervention entscheidet. Das *Engagement der Einrichtungsleitung* während der Umsetzung umfasste koordinative Aufgaben, z. B. Terminabsprachen oder Verteilung von Aufgaben unter den Individuen. Die Einrichtungsleitung war Ansprechperson sowohl für die Projektinitiatoren als auch die Fachpersonen in den Schulen und Kitas. Außerdem stellte sie Strukturen und Ressourcen in der Einrichtung für die GF bereit.

Die *inhaltliche Platzierung der Intervention* bezeichnet die Vereinbarkeit von Interventionsthemen mit Aktivitäten oder inhaltlichen Schwerpunkten von Einrichtungen. Schwierigkeiten traten auf, wenn die Interventionen nicht mit dem Setting und seinen übrigen pädagogischen Aktivitäten kompatibel waren und sich nicht in eine bestehende Präventionskultur einfügten (Z13).

Die *Bereitschaft der Einrichtung* bezeichnet ihre offene Grundhaltung gegenüber einer Intervention. Sie förderte in den Einrichtungen das initiale Engagement und die Aufrechterhaltung von Interventionen. Wenn bereits ähnliche Interventionen verfolgt wurden, war der Weg für neue Interventionen gebahnt (Z14). Dennoch bestand die Gefahr, dass sie aufgrund begrenzter Ressourcen als konkurrierende Angebote in den Einrichtungen wahrgenommen wurden.

#### Outer-Setting-Ebene

In den Schulen war die *Vereinbarkeit mit dem Bildungsauftrag* von Bedeutung, die im Allgemeinen die Kompatibilität mit dem Lehrplan und im Speziellen jene mit Unterrichtsfächern betrifft (Z14). Verknüpfungsstellen von Interventionen wurden von 2 Lehrkräften mit den Fächern Religion und Sport beschrieben. Durch die Interventionsteilnahme ergab sich der Vorteil, dass das Lehrangebot um Inhalte ergänzt wurde, die im Curriculum nicht (ausreichend) berücksichtigt worden waren. Dadurch dass sowohl in den Kitas als auch in den Grundschulen GF zum Teil nicht als mit dem Bildungsauftrag vereinbares Aufgabenfeld betrachtet wurde, war ihre Durchführung erschwert. Es entstand der Eindruck, dass Fachpersonen Bildungsaufgaben priorisierten (Z15). Es wurde auch eine stärkere politische Unterstützung von GF durch eine Priorisierung im Schulcurriculum gefordert.

Äußerungen zu *rechtlichen Rahmenbedingungen* betrafen neben Gesetzesvorgaben vor allem pandemiebedingte Auflagen, die Interventionen zur GF mehrheitlich in ihrer Umsetzbarkeit einschränkten (Z16).

Das Engagement der Befragten wurde auch von *politischen Handlungen und Entscheidungen* beeinflusst. Beispielsweise veranlasste die Reduktion der Sportstunden in niedersächsischen Grundschulen dazu, *fit für pisa* *+ *zu initiieren.

Für 2 Befragte war die Finanzierbarkeit von Bedeutung. Eine gesicherte *Finanzierung* stand begrenzten finanziellen Mitteln gegenüber.

#### Handlungsempfehlungen

In der Präventionsforschung ist es bedeutsam, Ergebnisse für die Praxis zugänglich zu machen [16, 31]. Hierzu wurden auf Basis der identifizierten Gelingens- und Hindernisfaktoren 22 praxistaugliche Handlungsempfehlungen (Tab. 4) formuliert.

**Tab. 4:** Datenbasierte Handlungsempfehlungen für die Implementierung von Projekten zur Gesundheitsförderung (GF), orientiert am Konzept CFIR (Consolidated Framework for Implementation Research [19])

CFIR-Ebene	Handlungsempfehlungen
*Individuum:* Projektbeteiligte Fachpersonen	Exploration und Förderung der Motivation projektbeteiligter Fachpersonen in Kitas und Grundschulen für die GF
	Befähigung der Fachpersonen in Kitas und Grundschulen zur selbstständigen Umsetzung von GF
	Nutzung vorbestehender Fähigkeiten und Kompetenzen der Fachpersonen in Kitas und Grundschulen
*Intervention:* Projekte zur GF in Kitas und Grundschulen	Erläuterung der Zielsetzung und Sinnhaftigkeit seitens der Projektinitiatoren
	Teilen von Erkenntnissen, Erfahrungen oder Evidenzen zu den Wirkungen eines Projekts
	Kontinuierliche Überprüfung der Akzeptanz eines Projekts seitens der projektbeteiligten Fachpersonen, der Eltern und der Kinder (Zielgruppe)
	Erstellen eines Handlungsplans, um die Projektumsetzung zu fördern
	Bereitstellen von Informationsquellen zu den Projekten
	Implikation von Möglichkeiten zur flexiblen Umsetzung von Projektinhalten
*Prozess: *Kooperation von Projektinitiatoren und projektbeteiligten Fachpersonen in den Einrichtungen Kitas und Grundschulen	Vorantreiben eines multiprofessionellen Vorgehens
	Etablierung, Förderung und Nutzung von Kommunikationsformen und -wegen
	Förderung von Wertschätzung und Aufbau eines Teamgefühls zwischen Projektinitiatoren und projektbeteiligten Fachpersonen
	Aufbau von Arbeitsbeziehungen auf Augenhöhe
	Abstimmung und Koordination von Prozessen in der Zusammenarbeit
	Aktive Beteiligung aller projektbeteiligten Fachpersonen
	Kontinuierliche Evaluation der Projekte
*Inner Setting: *Kitas und Grundschulen als Umsetzungsorte der GF	Förderung der aktiven Beteiligung der Einrichtungsleitung
	Überprüfung, Nutzung und Ausbau vorhandener Ressourcen
	Anknüpfen an bereits bestehende Bemühungen zur GF in den Einrichtungen
	Betrachten der Umsetzung von Projekten zur GF als Teamaufgabe in Einrichtungen
*Outer Setting: *Gesellschaftliches, rechtliches und kulturelles Umfeld der Projekte	Schaffen von Rahmenbedingungen für den Austausch und die Vernetzung von projektbeteiligten Personen
	Vereinbarkeit von Projekten mit äußeren Rahmenbedingungen

### Auswirkungen der COVID-19-Pandemie

Anhand von 69 Interviewpassagen wurden positive und negative Auswirkungen der COVID-19-Pandemie auf die Durchführung von Interventionen zur GF analysiert.

Anhand von 4 Interviewaussagen konnte die Vereinbarkeit einer Intervention mit den Hygieneregeln als neuer Gelingensfaktor identifiziert werden (Z17). Auch die Anpassungsmöglichkeit von Interventionen ist in der COVID-19-Pandemie als Gelingensfaktor zu bewerten (siehe Interventionsebene, *Handlungsspielräume* und *Anpassungen*). Dabei bekam GF einen höheren Stellenwert, wenn Interventionen im Unterschied zu anderen Angeboten in der Pandemie fortgesetzt werden konnten. Sie stellten eine Abwechslung in den LW dar und wurden teilweise als exklusive Möglichkeit betrachtet, Bewegungsförderung weiterhin anbieten zu können (Z17). Während der COVID-19-Pandemie wurde das Thema Gesundheit und GF bewusster wahrgenommen.

Nichtsdestotrotz war zu verzeichnen, dass die GF während der COVID-19-Pandemie, z. B. aufgrund reduzierter zeitlicher Ressourcen, an Stellenwert verloren hatte (Z18). Die Durchführung der Inventionen wurde teilweise aufgrund von reduzierten Ressourcen in Krankheits‑/Quarantänezeiten sowie allgemeiner Mehrbelastung in den LW unterbrochen (Z18), was sich hinderlich auf die Kooperation von Individuen auswirkte.

Eine abschließende Beurteilung der COVID-19-Pandemie als förderlicher oder hinderlicher Faktor war in dieser Studie nicht möglich.

## Diskussion

Mithilfe des CFIR wurden 22 Faktorengruppen analysiert. Eine positive Haltung und Überzeugungen sowie Motivation für eine Intervention waren auf der Individuenebene entscheidend. Weitere bedeutsame Faktoren für eine erfolgreiche Implementierung waren einerseits die Akzeptanz und angemessene Komplexität einer Intervention, eine gelungene Kooperation aller Beteiligten sowie die Vereinbarkeit mit Einrichtungsstrukturen. Im Outer Setting war bedeutsam, dass Interventionsinhalte mit dem Bildungsauftrag kompatibel waren.

Im Gleichklang mit Schrader et al. [32] wurden vorrangig Gelingensfaktoren identifiziert. Die Stärke dieser Studie liegt in der umfassenden Exploration implementierungsrelevanter Faktoren auf der Individuenebene, was auf das Studiendesign zurückzuführen ist. Projektbeteiligte Fachpersonen zu befragen, wird als sinnvoll erachtet, um Implementierungsfragen zu beforschen [33]. In Folgestudien sollten die Inner-Setting- und Outer-Setting-Ebene stärker berücksichtigt werden, was aufgrund methodischer Einschränkungen in dieser Studie nicht gelungen ist. Eine Chance besteht darin, die Stichprobe auf Projektinitiatoren auszuweiten.

Inhaltliche Parallelen der Ergebnisse dieser Studie zu den identifizierten Implementierungsfaktoren anderer Forschender [7, 34] unterstreichen die Relevanz der Ergebnisse. Obwohl die qualitative Erhebung mit einer Berücksichtigung von 4 Projekten zur GF in 3 Kitas und 12 Grundschulen in einer Region keine Generalisierbarkeit der Ergebnisse für sich beanspruchen kann [35], kann eine gewisse Übertragbarkeit der Ergebnisse in andere Kontexte der GF aufgrund der Übereinstimmung der Ergebnisse angenommen werden. Anschlussstudien, die andere methodische Ansätze, wie z. B. Mixed Methods nutzen, könnten auf der Vorarbeit dieser Studie aufbauen. Die Kombination verschiedener Erhebungsmethoden diente in dieser Studie nicht einer Triangulation, was eine Limitation darstellen könnte.

Die Ergebnisse leisten ferner einen Beitrag in der Fidelity-Adaptation-Debatte [13] der Implementierungswissenschaften, in der Anpassungen und die Implementierungstreue von Interventionen kontrovers diskutiert werden. In der zum Untersuchungszeitpunkt gegenwärtigen COVID-19-Pandemie waren Anpassungen und Handlungsspielräume Erfolgsfaktoren und entsprachen notwendigen Voraussetzungen, die die Umsetzung von GF erst ermöglichten. Der Erfolgsfaktor *Anpassungen* sollte zukünftig verstärkt im Implementierungskontext einer Intervention erforscht werden. Inwiefern sich Umsetzungsgüte bzw. -treue und Adaptationen bei der Implementierung integrieren lassen könnten, kann auch diese Studie nicht beantworten.

In der Präventionsforschung ist es wichtig, wissenschaftliche Ergebnisse der Praxis zugänglich zu machen [16, 31]. Zu diesem Zweck wurden Handlungsempfehlungen formuliert, die Orientierungshilfen bei der Planung, Durchführung und Evaluation von GF in der Praxis bieten. Aufgrund ihrer allgemeinen Formulierung können sie nach Anpassungen auch in spezifischen Kontexten verwendet werden.

Obwohl das CFIR Beziehungen zwischen Faktoren und Ebenen nicht ausreichend aufzeigen kann [24], zeichneten sich in der Analyse Zusammenhänge zwischen der Individuen- und Interventionsebene ab, die auch Hartmann et al. beschreiben [36]. Es zeigte sich an zahlreichen Stellen eine inhaltliche Nähe zwischen einzelnen Faktoren(gruppen) und Ebenen. In zukünftigen Studien sollten die Interaktion und Beziehung zwischen Faktoren stärker fokussiert werden, um besser zu verstehen, wie sich Faktoren positiv und negativ beeinflussen. Dies könnte unter Zuhilfenahme weiterer Rahmenmodelle gelingen, die außerdem die Validität der Ergebnisse stärken könnten.

Schrader et al. merken in ihrem Review zur Implementationsforschung im Bildungssystem beispielsweise die fehlende Orientierung an Frameworks kritisch an [32]. Die Verwendung des CFIR in der Analyse kann daher als methodische Stärke dieser Studie bewertet werden. Insgesamt manifestierte sich die Eignung des CFIR darin, eine Mehrebenenperspektive zu eröffnen. Möglicherweise könnte die theoretische Verankerung der Ergebnisse gesteigert werden, wenn das CFIR bereits während der Datensammlung und -auswertung mittels QIA berücksichtigt wird [20].

Eine besondere Stärke der Studienplanung und -durchführung liegt in der Zusammenarbeit von Wissenschaft und Praxis, die in der Präventionsforschung gleichermaßen gefordert wie notwendig ist [15]. Experten*innen der Gesundheitsregion Göttingen/Südniedersachsen beteiligten sich an der Auswahl der Projekte für diese Erhebung und ermöglichten den Zugang zum Forschungsfeld, in dem sie wichtige Informationen mit dem Studienteam teilten. Im Sinne einer partizipativen Forschung könnte es sinnvoll sein, Praktiker*innen auch in die Datenanalyse einzubeziehen.

Da diese Studie während der COVID-19-Pandemie durchgeführt wurde, konnten pandemiebedingte Einflussfaktoren herausgestellt werden. Erhebungen zu pandemiefreien Zeitpunkten könnten eine sinnvolle Ergänzung sein, die vergleichende Überlegungen zulassen und neue Ergebnisse hervorbringen könnten.

Eine Limitation dieser Arbeit ist, dass Gelingens- und Hindernisfaktoren nicht im gleichen Ausmaß identifiziert werden konnten. Letztere wurden nur vereinzelt auf der Individuen‑, Inner-Setting- und Prozessebene sichtbar, was auf die positive Grundhaltung und hohe Motivation der Interviewpersonen für das Themenfeld GF zurückzuführen sein könnte. Es ist von einer Verzerrung zugunsten positiver Implementierungsfaktoren auszugehen, die die gesamte Analyse durchzieht. Um dieser Schwachstelle vorzubeugen wurde versucht, Interviewpersonen zu gewinnen, die sich gegen die Teilnahme an einem der 4 Projekte entschieden hatten, allerdings ohne Erfolg.

Eine weitere Limitation besteht darin, dass die Konstrukte des CFIR in der Analyse unberücksichtigt blieben, sodass sein Potenzial – auch aufgrund fehlender Berücksichtigung in der Datenerhebung – nicht ausgeschöpft werden konnte. Um die Ergebnisse stärker theoretisch zu verankern, ist es unerlässlich, die Konstrukte miteinzubeziehen [20]. Gleichwohl leistete diese Studie eine wichtige Vorarbeit, um in Folgestudien eine umfassende Operationalisierung der CFIR-Ebenen vorzunehmen.

## Fazit

Diese qualitative Studie liefert einen Beitrag für die Präventions- und Implementierungsforschung in dem Teilbereich Gesundheitsförderung (GF) in den Lebenswelten (LW) Kita und Grundschule. Die identifizierten Faktoren weisen darauf hin, wie die Umsetzung in diesen LW in Deutschland zukünftig vorangetrieben werden kann. Elementare Voraussetzung für die Entwicklung einer Präventionskultur, die GF als integralen Bestandteil unterstützt, sind motivierte Individuen, deren Engagement durch bereitgestellte Ressourcen ermöglicht und von Projektinitiatoren unterstützt wird. Das Ergebnis, dass sich Fachpersonen in Kitas und Grundschulen durch ein hohes Problembewusstsein für das Thema Gesundheit im Kindesalter und ein hohes Engagement für das Feld der GF auszeichnen, obwohl diese Aufgabe teilweise in Konkurrenz zum Bildungsauftrag wahrgenommen wurde, sollte zur weiteren politischen Unterstützung veranlassen. Es bleibt offen, wie man die Rahmenbedingungen für die Implementierung von GF optimieren kann, um das Engagement in den Bildungseinrichtungen stärker zu fördern. Diese Studie verdeutlicht, wie wichtig es ist, die Praxisperspektive bei der Implementierung von Interventionen zur GF zu berücksichtigen. Die Auswirkungen der COVID-19-Pandemie zeigen eindrücklich, wie bedeutsam der Implementierungskontext für eine erfolgreiche GF ist.

### Supplementary Information

Onlinematerial 1: Interviewleitfaden und Fokusgruppen-Inputs

Onlinematerial 2: Kategoriensystem, einschließlich Anzahl der Kodierungen und Kodierleitfaden
