# Supplementary material for: Facilitators and barriers to the implementation of health promotion in daycare centers and elementary schools based on four selected projects
Source: Bundesgesundheitsblatt Gesundheitsforschung Gesundheitsschutz. 2024 Aug 20;67(9):1021–30. [Article in German] doi: 10.1007/s00103-024-03935-0 (PMC11349828; doi:10.1007/s00103-024-03935-0)
Supplement: Supplementary file 1 — Onlinematerial 1: Interviewleitfaden und Fokusgruppen-Inputs [file 103_2024_3935_MOESM1_ESM.pdf]

Onlinematerial 1: Interviewleitfaden und Fokusgruppen-Inputs

Interviewleitfaden

1. Sie nehmen an [Programmname] als [Funktion] teil.
Erzählen Sie mir doch bitte davon, wie Sie zu [Programmname] gekommen sind.

- Projektziele
- Unterstützung durch die vorhandene Struktur der Organisation

• Wie sind Sie auf [Programmname] aufmerksam geworden?
• Inwiefern sahen Sie vor der Einführung des Programms [*Name*] einen Bedarf für die Intervention?
• Wie haben Sie sich auf [Programmname] vorbereitet?

2. Erzählen Sie mir bitte, was Sie antreibt, sich für das Thema Gesundheitsförderung in Ihrem beruflichen Tätigkeitsfeld zu engagieren.

- Motivationsgründe
- Nutzung eigener Kompetenzen
- Persönlicher Unterstützungsbedarf

• Was hat Sie motiviert, sich für das Projekt zu engagieren?
• Inwiefern waren Ihr beruflicher Hintergrund und Ihr Vorwissen für Sie im Projekt hilfreich?

3. Erzählen Sie mir doch bitte ausführlich von Ihren persönlichen Erfahrungen mit [Programmname].

- Anzeichen positiver Auswirkungen
- Bedeutung des Projekts für den Alltag der Kinder
- Schwierigkeiten der Implementierung
- Austausch mit Erziehungsberechtigten
- Akzeptanz im Kollegium

• Wie erleben Sie die Kinder während der Intervention?
• Was war bisher für Sie die schönste Erfahrung im Rahmen des Projektes?
• An welche besonderen Erlebnisse denken Sie im Zusammenhang mit [Programmname]?
• Wie erleben Sie die elterliche Einstellung zu der Intervention?
• Inwieweit erleben Sie Hemmnisse in Ihrem Team bei der Umsetzung des Programmes?
• Inwieweit erleben Sie Hemmnisse bei sich selbst bei der Umsetzung des Programms?

4. Bitte erzählen Sie mir von der Zusammenarbeit der einzelnen Beteiligten, die beruflich an [Programmname] mitwirken.

- Begleitung der Einführung
- Kommunikation der Akteur*innen
- Informationsquellen
- Funktion der Schul- oder Kita-Leitung
- Team- vs. Einzelaufgabe

• Welche Hilfestellungen und Informationen haben es erleichtert, die Intervention einzuführen und umzusetzen?
• Inwiefern wurden Ihnen Begleitmaterialien zur Durchführung der Intervention bereitgestellt?
• Wie funktioniert die Zusammenarbeit mit Projektbeteiligten innerhalb Ihrer Einrichtung?
• Welche Bedeutung hat die Zusammenarbeit zwischen einzelnen Berufsgruppen bei der Umsetzung des Projektes?
• Inwiefern haben Sie konkrete Ansprechpartner*innen des Projektes außerhalb Ihrer Einrichtung?
• Inwieweit sind Ihnen diese Ansprechpartner*innen für das Projekt persönlich bekannt?

5. Erzählen Sie doch bitte, inwieweit Sie sich an Entscheidungen zu dem Projekt beteiligen können.
- Entscheidungsprozesse
- Ausgestaltung des Projektes
• Inwiefern können Sie eigene Ideen in das Programm einfließen lassen?
• Inwiefern können Sie sich an der Gestaltung des Programms beteiligen?

6. Wie ließ sich das Projekt in Ihren Arbeitsalltag integrieren?
- Eignung der örtlichen Gegebenheiten (Raum, Zeit)
- Auswirkungen auf die Arbeit
- Auswirkungen der Pandemie auf das Projekt
- Übertragbarkeit auf andere Organisationen
• Wie hat sich die Coronapandemie auf die Umsetzung des Projektes ausgewirkt?
• Wie schätzen Sie die Chancen ein, dass das Projekt nach der Pandemie wieder weitergeführt wird?
• Wie schätzen Sie die Übertragbarkeit des Projekts auf andere Einrichtungen ein?

7. a) Erzählen Sie mir doch bitte, wie Sie persönlich auf die Zukunft von [Programmname] schauen. b) Inwiefern gibt [Programmname] Anstöße für die Gesundheitsförderung auch über das Projektende hinaus? (NF: Wie wirkt sich das Programm für die Gesundheit von Kindern auch nach Projektende in Ihren Augen aus?)
- Wünsche
- Beiträge zur Weiterentwicklung
- Tipps als Expert*in für die Intervention
• Wo sehen Sie persönlich Ansätze für die Weiterentwicklung des Projektes?
• Was müsste passieren, damit das Ihr „Traum"-Projekt wird?
• Wie können Sie persönlich positive Veränderungen anstoßen?
• Wie wirkt sich das Programm für die Gesundheit von Kindern auch nach Projektende in Ihren Augen aus?

8. Abschluss
Gibt es für Sie noch Punkte, die bisher nicht angesprochen worden sind?
Gibt es Fragen, die Sie erwartet hätten und nicht gestellt worden sind?
Haben Sie noch Fragen?

➢ Vielen Dank für Ihre Teilnahme, ich stelle die Tonbandaufnahme nun ab.

Aufrechterhaltungsfragen
- Und weiter?
- Wie war das für Sie?
- Erzählen Sie doch ein wenig mehr darüber…
- Und dann?
- Wie ging es weiter?
- Was bringen sie mit … (noch) in Verbindung?

Fokusgruppen-Inputs

1. Was treibt Sie dazu an, sich für *fit für pisa +* einzusetzen?
2. Wie schätzen Sie die Zusammenarbeit im Rahmen von *fit für pisa +* ein?
3. Wie lässt sich *fit für pisa +* in Ihren Arbeitsalltag integrieren?
4. Wie wirkt sich die Corona-Pandemie auf *fit für pisa +* aus?
5. Welche Auswirkungen von *fit für pisa +* sehen Sie?
