# Supplementary material for: Facilitators and barriers to the implementation of health promotion in daycare centers and elementary schools based on four selected projects
Source: Bundesgesundheitsblatt Gesundheitsforschung Gesundheitsschutz. 2024 Aug 20;67(9):1021–30. [Article in German] doi: 10.1007/s00103-024-03935-0 (PMC11349828; doi:10.1007/s00103-024-03935-0)
Supplement: Supplementary file 2 — Onlinematerial 2: Kategoriensystem, einschließlich Anzahl der Kodierungen und Kodierleitfaden [file 103_2024_3935_MOESM2_ESM.pdf]

Onlinematerial 2: Kategoriensystem einschließlich Anzahl der Kodierungen und Kodierleitfaden

**Bezeichnung der Hauptkategorie (Anzahl der Codes)**<br>▪ **Bezeichnung der Unterkategorie (Anzahl der Codes)**	**Kategoriedefinitionen, Ankerzitate & Abgrenzungshinweise**<br><br>*Anmerkung: Jede Hauptkategorie wurde definiert. Bei den Unterkategorien wurde bis auf wenige Ausnahmen von einer Definition abgesehen. Die Referenzen für die Ankerzitate liegen der Erstautorin vor.*
Erstkontakt der Interviewperson (IP) mit dem Projekt (30)	Definition:<br>Die Hauptkategorie trifft zu, wenn die IP darüber berichtet oder erzählt, wie sie von dem Projekt erfahren hat oder initial der Kontakt mit Projektanbietenden oder Projektinhalten entstanden ist. Hierzu zählen mündliche oder schriftliche Ansprachen der IP.<br><br>Ankerzitat:<br>*„Als ich hier an diese Schule kam, WAR das eben schon etabliert. Und ähm seitdem ich hier bin, wird das eben durchgeführt und wir haben jetzt nur positive Erfahrungen und da habe ich das tatsächlich gar nicht weiter hinterfragt. Sondern wir haben es einfach umgesetzt, weil es eben schon so gut etabliert gewesen ist. Durch frühere Sportkollegen und Schulleitung und ähnliches."*
▪ Informationen durch Projektorganisation oder -initiatoren (9)	Definition:<br>Die Unterkategorie wird kodiert, wenn die IP aufgrund von Informationen der Projektorganisation oder -initiatoren von dem Projekt erfahren hat.<br><br>Ankerzitat:<br>*„Und dann kam das Projekt ja auch hier in in die KITa. Und die haben sich dann vorgestellt und haben erzählt, was sie so machen, was sie sich vorstellen. Ja. Und letztendlich ähm es war ja eigentlich gar keine schwere Entscheidung. Das war so, dass wir gesagt haben 'Ja KLAR machen wir mit´. (.)“*
▪ Kennenlernen des Projekts in der Einrichtung (16)	Definition:<br>Die Unterkategorie ist zutreffend, wenn die IP davon erzählt, wie sie in der Einrichtung (Kita oder Schule) von dem Projekt erfahren hat. Wenn die IP von anderen Einrichtungsmitarbeitenden Informationen zu dem Projekt erfahren hat oder das bereits umgesetzte Projekt praktisch kennen gelernt hat, wird diese Unterkategorie kodiert.<br><br>Ankerzitat:<br>*„ÄHM und dann hat sie halt nur gesagt, dass dann IRgendwann sone Sportlerin, ich weiß ihren Namen nicht mehr, an die Schule (.) kommen soll und ob ich dann den Sporttag organisiere. (..)“*
▪ Zugangswege unabhängig von Initiatoren oder Einrichtungen (5)	Definition:<br>Die Unterkategorie ist zutreffend, wenn die IP nicht vordergründig in der Schule oder Kita von dem Projekt und nicht über Informationen der Projektorganisatoren oder -initiatoren von dem Projekt erfahren hat.<br><br>Ankerzitat:<br>*„(..) Ähm ich habe es zuHAUse hier gefunden. ÄHM da mein Mann es aus der Schule mitgebracht hat. (...)“*
Meinung der IP zum Bedarf des Projekts (46)	Definition:<br>Die Hauptkategorie trifft zu, wenn die IP ihre Auffassungen und Meinungen zur Notwendigkeit und dem Bedarf des Projektes in/für die Zielgruppe äußert.<br><br>Ankerzitat:<br>*„Also schon denk ich hoch ähm. Speziell auch jetzt wäre es wahrscheinlich auch ähm WÜNschenswert, jetzt gerade in Corona-Zeiten. Ich habe ähm damals bei der Klasse, die waren eigentlich alle relativ fit, bis auf ein zwei KINder, ähm da hätte ich jetzt vielleicht nicht gesagt, die müssten jetzt unbedingt mehr machen.“*
▪ Beschreibung der Bedürftigkeit in Bezug auf die Zielgruppe (23)	Definition:<br>Die Unterkategorie ist zutreffend, wenn die IP anhand der Bedürftigkeit der

**Bezeichnung der Hauptkategorie (Anzahl der Codes)**<br>▪ **Bezeichnung der Unterkategorie (Anzahl der Codes)**	**Kategoriedefinitionen, Ankerzitate & Abgrenzungshinweise**<br><br>Anmerkung: *Jede Hauptkategorie wurde definiert. Bei den Unterkategorien wurde bis auf wenige Ausnahmen von einer Definition abgesehen. Die Referenzen für die Ankerzitate liegen der Erstautorin vor.*
	Zielgruppe den Bedarf für die Umsetzung des Projekts ableitet. Die Unterkategorie wird auch kodiert, wenn die IP einen über die Zielgruppe hinausgehenden Bedarf für das Projekt beschreibt. Auch die Erläuterung einzelner bedürftiger Zielgruppenmitglieder wird in der Unterkategorie kodiert.<br><br>Ankerzitat:<br>„Ich glaube es gibt nen TEIL der KINder, die brauchen fit für pisa nicht. JA fünfzig Prozent oder vierzig Prozent der KINder, die haben von ZUhause sind die so sportgefördert und werden gesund ernährt, die brauchen das nicht. Aber die ANderen fünfzig Prozent, die werden für ihr Leben profitieren können. Das ist jedenfalls meine MEInung.“
▪ Allgemeine Einschätzung über die Höhe des Bedarfs (15)	Definition:<br>Die Unterkategorie trifft zu, wenn die IP allgemeine Aussagen darüber trifft, inwiefern ein Bedarf oder seine Höhe ausgeprägt ist. Indikatoren können Adjektive wie zum Beispiel niedrig, hoch und wichtig sein.<br><br>Ankerzitat:<br>*„(.) Ähm grundsätzlich ist es glaube ich sehr, sehr hoch. Wir haben hier den Vorteil, dass es einfach schon immer so war. Und Sportfreundliche Schule ist schon ewig und wir auch schon vieles machen. Aber ich weiß eben auch von anderen, da war es ganz neu, dass man einfach feste BesSTANDteile auch im Tagesablauf eben in der Schule hat, die dann wirklich eingehalten werden.“*
▪ Überlegungen zur Bedarfsabschätzung (8)	Definition:<br>Die Unterkategorie trifft zu, wenn die IP erzählt, woran sie einen Bedarf der Zielgruppe festmacht. In dieser Unterkategorie kann die IP auch Zweifel oder Unsicherheiten zum Ausdruck darüber bringen, ob sie einen Bedarf der Zielgruppe sehen oder bewerten kann.<br><br>Ankerzitat:<br>*„(..) VOR der Einführung (6), dass die da, weiß ich nicht, kann ich jetzt gar nicht so beantworten. Habe ich Bedarf gesehen? Ich kannte Fit fürs Leben nicht. Von DAher, wenn ich es nicht KENNE, weiß ich ja nicht, ob ich da Bedarf habe oder nicht. Das könnte ich nicht beantworten. Ich würde jetzt mal sagen, ich habe keinen BeDARF gesehen, weil mir diese ganze Aktion ja unbekannt war. (.)“*
Vorbereitung der IP auf die Projektumsetzung (27)	Definition:<br>Die Hauptkategorie trifft zu, wenn die IP davon erzählt, welche Schritte und Maßnahmen sie in Vorbereitung auf die Umsetzung des Projekts selbst ergriffen hat und welche Hilfestellungen und Informationen sie empfangen hat.<br><br>Ankerzitat:<br>*„Und dann habe ich eine kleine EINführung bekommen (..). Ähm von (.) Ach ich weiß nicht mehr. Weiß ich nicht mehr wie ER heißt. Jemand auch [von der Projektorganisation], der das schon länger durchführt. Der hat uns dann so Tipps gegeben (.), WEIL es ja auch drau/ Es sollte ja auch draußen stattfinden und manche Sachen, die man / Man hatte halt son Leitfaden. Und manche Dinge musste man halt anders umsetzen draußen, weil man die Geräte nicht so hatte wie in der HALLE. Und da haben wir dann halt TIPPS bekommen oder konnten uns austauschen, wie können wir bestimmte Geräte ähm erSETzen. Ja, das war eigentlich so die erste Vorbereitung.“*
▪ Selbstständige Vorbereitung (6)	Definition:<br>Die Unterkategorie trifft zu, wenn die IP beschreibt, wie sie sich auf die Interventionsumsetzung eigenständig vorbereitet hat. Darunter fällt die eigenständige Suche nach Informationen, die Beschaffung von Sachmitteln oder auch der Austausch mit Kolleg*innen innerhalb einer Einrichtung. Insgesamt handelt es sich um eine Vorbereitung der IP, die nicht durch die Projektinitiator*innen vorgenommen wird.
▪ Strukturelle Maßnahmen in der Einrichtung (4)	Definition:<br>Die Unterkategorie trifft zu, wenn die IP erläutert, welche strukturellen Schritte sie in der Einrichtung, d. h. Schule oder Kita vor der Projektumsetzung eingeleitet hat.

Bezeichnung der Hauptkategorie (Anzahl der Codes)<br>▪ Bezeichnung der Unterkategorie (Anzahl der Codes)	Kategoriedefinitionen, Ankerzitate & Abgrenzungshinweise<br><br>Anmerkung: *Jede Hauptkategorie wurde definiert. Bei den Unterkategorien wurde bis auf wenige Ausnahmen von einer Definition abgesehen. Die Referenzen für die Ankerzitate liegen der Erstautorin vor.*
	Dabei kann die IP beispielsweise auf Zuständigkeiten, Einrichtungskonzepte oder Absprachen mit Vorgesetzten hinweisen.<br><br>Abgrenzung:<br>- Unterkategorie *Vereinbarkeit mit strukturellen Bedingungen* (Hauptkategorie *Integrierbarkeit des Projekts in der Einrichtung*)<br>In dieser Unterkategorie werden Aussagen der IP kodiert, die die Vereinbarkeit des Projekts während der Umsetzungsphase mit den Bedingungen (räumlich, örtlich, materiell, zeitlich) in der Einrichtung betreffen.<br><br>Ankerzitat:<br>*„Also dass die Schulleitung vorbereitet ist UND ähm dann könnte man sich einfach ne das in einem (.) in ein THEma einbetten, also in eine Unterrichtseinheit. (...)“*
▪ Wissensvermittlung durch das Projektteam (13)	Definition:<br>Die Unterkategorie trifft zu, wenn die IP mündlich oder schriftlich zum Beispiel mithilfe von Informationsmaterialien durch die Projektinitiatoren informiert worden ist.<br><br>Abgrenzung:<br>- Hauptkategorie *Zusammenarbeit der IP mit anderen Projektbeteiligten*<br>In dieser Hauptkategorien werden Erfahrungen der IP mit der Zusammenarbeit im Rahmen der Projektumsetzung kodiert, sie betreffen nicht die vorbereitenden Maßnahmen für die IP durch das Projektteam.
▪ Meinungen zur Notwendigkeit von Vorbereitungen (4)	Definition:<br>Die Unterkategorie ist zutreffend, wenn die IP erzählt, ob und welche Vorbereitungen für die Projektumsetzung erforderlich sind. Die Unterkategorie ist auch zutreffend, wenn die IP benennt, dass die Projektumsetzung keine Vorbereitung erfordert.<br><br>Ankerzitat:<br>*„Hm (überlegend) also vorbereitet haben wir uns eigentlich NICHT wirklich daRAUF, denn es wurde uns ja geSAGT äh 'Es kommen dann (.) die LEUte, die dieses Projekt da durchFÜHren ZU uns und führen das dann mit den KINdern durch. Ja. (.) Und WIR WAren im Prinzip dann die HELfenden Hände dabei. Ja. (.) Ja so war das.“*
Einsatz von eigenen Kompetenzen der IP (59)	Definition:<br>Die Hauptkategorie trifft zu, wenn anhand der Aussagen der IP deutlich wird, wie auf die IP auf ihre persönlichen Kompetenzen zurückgreifen und sie während der Projektumsetzung einbringen konnte. Dies kann sich sowohl in der Haltung der IP, ihrer Anwendungsbereitschaft, d.h. Handlungskompetenz und in ihrer Nutzung von Vorwissen für die Umsetzung des Projekts zeigen.<br><br>Abgrenzung:<br>- Hauptkategorie *Engagement der IP für die* Gesundheitsförderung<br>Unter dieser Hauptkategorie wird die grundsätzliche Haltung der IP kodiert, sich mit den Themen Gesundheitsförderung und Prävention im Rahmen der Berufstätigkeit zu beschäftigen. Hierbei geht es nicht um Aussagen, die sich direkt auf die in der Studie untersuchten Präventionsmaßnahmen beziehen.<br><br>Ankerzitat:<br>*„Und als ich dann damit angefangen hab ist es dann einfach gewachsen. Dann WUSste man SCHON, was kann man wie wo EINsetzen um die einzelnen Fertigkeiten noch spezieller zu fördern. Oder was macht den Kindern SPASS und wo kann man da weiter ansetzen (.). Das hat sich dann so im Laufe des Projekts auch entwickelt, wie man das am besten macht (.).“*
▪ Handlungskompetenz und Anwendungsbereitschaft (28)	Definition:<br>Die Unterkategorie wird kodiert, wenn die Handlungsfähigkeit der IP deutlich wird. Die Handlungsfähigkeit ist im Allgemeinen als Überbegriff von Sozial-, Fach- und Methodenkompetenz zu verstehen. Äußerungen und Erzählungen, die auf die Handlungs- und Anwendungsfähigkeit der IP bei der praktischen Umsetzung von

**Bezeichnung der Hauptkategorie (Anzahl der Codes)**<br>▪ **Bezeichnung der Unterkategorie (Anzahl der Codes)**	**Kategoriedefinitionen, Ankerzitate & Abgrenzungshinweise**<br><br>Anmerkung: *Jede Hauptkategorie wurde definiert. Bei den Unterkategorien wurde bis auf wenige Ausnahmen von einer Definition abgesehen. Die Referenzen für die Ankerzitate liegen der Erstautorin vor.*
	Projektinhalten hinweisen, werden unter dieser Unterkategorie kodiert. Dies kann zum Beispiel anhand von Äußerungen der IP zum Umgang mit Schwierigkeiten im Rahmen des Projekts deutlich werden.<br><br>Abgrenzung:<br>Unterkategorie *Haltung gegenüber der Ausgestaltung des Projekts* (Hauptkategorie *Einsatz von eigenen Kompetenzen der IP)*<br>Diese Unterkategorie beinhaltet grundlegende Verhaltensweisen, die auf die Haltung der IP gegenüber Projektinhalten schließen lassen. Sie beinhaltet hingegen keine Tätigkeiten während der Projektumsetzung.<br><br>Ankerzitat:<br>*„(..) Also im Religionsunterricht spricht man eben auch über traurig sein, über krank sein, über TOD. Und ähm ja in diesem Rahmen könnte man eben die Kinder erZÄHlen lassen, was Sie so erlebt haben, was sie TRAUrig macht. Wenn sie es nicht erzählen WOLLEN, kann man das MALEN oder SCHREIben lassen. (.) Ähm oder mit nem Freund drüber sprechen lassen beim Spaziergang übern Schulhof oder sowas. Und ähm JA so kann man irgendwie versuchen, das son bisschen zu erARbeiten. (...)“*
▪ Persönliche Haltung gegenüber der Ausgestaltung des Projekts (18)	Definition:<br>Die Unterkategorie wird verwendet, wenn sich anhand der Aussagen der IP eine Haltung der IP gegenüber bestimmten Projektinhalten ableiten lassen kann. Dies können Aussagen über Annahmen, Verhaltensweisen aber auch Gefühle der IP im Zusammenhang zu Projektinhalten sein.<br><br>Abgrenzung:<br>- Unterkategorie *Handlungskompetenz und Anwendungsbereitschaft* (Hauptkategorie *Einsatz von eigenen Kompetenzen durch die IP*)<br>In dieser Unterkategorie geht es um bestimmte Tätigkeiten der IP, die die Handlungsfähigkeit und -kompetenzen für die Projektumsetzung durch die IP transportieren. Es geht aber nicht um grundsätzliche Verhaltensweisen, die auf die Haltung der IP zu Projektinhalten schließen lassen.
▪ Einbringen von (Vor-)Wissen für das Projektengagement (13)	Definition:<br>Die Unterkategorie trifft zu, wenn die IP erklärt, wie sie eigene Vorkenntnisse und Fähigkeiten aus anderen Zusammenhängen in das Projekt einbringen konnte.<br><br>Ankerzitat:<br>*„Es geht ja eigentlich darum, diese ErZIEher und die Kinder ähm zu sensibiliSIEren oder ihnen das zu vermitteln, WELche Möglichkeiten hat man eigentlich im Walde und und was WArum ist das so gut DAS zu machen. Da hat mir natürlich die Vorbildung, WAS man also als Förster / Bin ja auch da Förster Förster studierter Förster. Weiß ich viel über den Wald, über die Pädagogik, weiß ich einiges über die Kinder. Und insofern hilft das natürlich alles zusammen da dann ein Programm zusammen zu STELlen, wo dann alle ihren SPASS haben. Sag ich jetzt mal son bisschen platt (..).“*
Engagement der IP für die Gesundheitsförderung (30)	Definition:<br>Die Hauptkategorie trifft zu, wenn die IP erläutert, was sie antreibt sich für das Feld der Gesundheitsförderung im Kontext ihrer beruflichen Tätigkeit einzusetzen. Dabei zeigt die IP unabhängig von dem jeweiligen Projekt, wie sie zu dem Feld der Gesundheitsförderung steht.<br><br>Ankerzitat:<br>*„Also ähm. Ich komme ja auch ausm heilpädagogischen Bereich (.). Und ich weiß einfach, wie wie WICHtig Bewegung ist in allen Hinsichten. Ob auf SPRAche, kognitive Fähigkeiten. Alles alles.“*
▪ Persönliche Erfahrungen (5)	
▪ Persönliche Überzeugungen und Annahmen (11)	
▪ Berufliches Selbstverständnis (14)	

**Bezeichnung der Hauptkategorie (Anzahl der Codes)**<br>▪ **Bezeichnung der Unterkategorie (Anzahl der Codes)**	**Kategoriedefinitionen, Ankerzitate & Abgrenzungshinweise**<br><br>Anmerkung: *Jede Hauptkategorie wurde definiert. Bei den Unterkategorien wurde bis auf wenige Ausnahmen von einer Definition abgesehen. Die Referenzen für die Ankerzitate liegen der Erstautorin vor.*
Motivationsgründe der IP für die Projektumsetzung (60)	Definition:<br>Die Hauptkategorie beschreibt die extrinsischen und intrinsischen Motivationsgründe der IP für die Durchführung konkreter Projektinhalte und ihre Teilnahme an einer Intervention. Auch von der IP wahrgenommene Unsicherheiten oder Bedenken gegenüber einer Intervention sind in dieser Hauptkategorie enthalten.<br><br>Abgrenzung:<br>- Hauptkategorie *Engagement der IP für die Gesundheitsförderung*<br>Diese Hauptkategorien bündelt Kodiereinheiten, die auf die Haltung der IP gegenüber den Themen Gesundheitsförderung und Prävention im Allgemeinen hinweisen, ohne dass die IP dabei Bezug auf eine Intervention nimmt.<br><br>Ankerzitat:<br>*„(.) Ich glaube die Motivation der KINder. Also ich denke, wenn man denen irgendwie dann erzählt so 'Da kommt jemand zu uns in Wald, um uns was zu erZÄHlen'. Zu sehen, wie die sich freuen (IP lacht) und was passiert, das freut einen dann ja auch ne. Macht einen dann selber irgendwie, dann hat man auch Lust dazu, wenn jetzt alle Kinder stöhnen würden und sagen würden 'Äh wieder kommt da jemand'. Dann wär es ein bisschen blöd. Aber auch so beim Sportabzeichen. Die haben da ja richtig LUST drauf, bringen alle ihre Sportsachen mit und wollen sich am liebsten schon morgens um acht ihre Sportsachen anziehen und so. Da ist man dann auch motiviert und hat Lust mitzumachen. (...)“*
▪ Einrichtungs- oder Gruppenzugehörigkeit (3)	Definition:<br>Wenn keine speziellen Gründe für oder gegen den Einsatz im Projekt genannt werden und die Teilnahme der IP an dem Projekt aufgrund der Zugehörigkeit zu einem Team oder einer Einrichtung, wo die Projektaktivitäten durchgeführt werden, stattfindet.
▪ Aussicht auf personelle Unterstützung in der Einrichtung (4)	
▪ Übernahme einer delegierten Tätigkeit (1)	
▪ Finanzieller Anreiz (1)	
▪ Persönliche Weiterentwicklung(5)	Definition:<br>Die Unterkategorie wird kodiert, wenn die IP sich ihre persönliche Weiterentwicklung und Anreize für ihre Berufsausübung durch die Projektteilnahme und -durchführung erhofft.
▪ Persönliche Überzeugungen und Annahmen (22)	
▪ Spaß an der Tätigkeit (5)	
▪ Persönliches inhaltliches Interesse (5)	Definition:<br>Die Unterkategorie wird kodiert, wenn die IP angibt, dass einzelne Themen sie für die Durchführung motiviert haben. Außerdem ist diese Unterkategorie zu kodieren, wenn die IP die inhaltliche Ausrichtung und Konzipierung der Intervention betont.<br><br>Ankerzitat:<br>*„(7) Was mich angetrieben hatte // (kurze Verzögerung durch Klärung der Kommunikation - zwei IPs wollten zeitgleich beginnen) // (3) WAS mich angetrieben hat ähm waren die verschiedenen Bereiche, dass die da zusammen ineinander gefasst haben. Das war einmal der SPORTliche Bereich, dass es noch einmal zusätzliche Sportstunden gegeben hat, was den Kindern IMmer guttut. „*
▪ Antizipierte Einstellungen gegenüber dem Projekt (14)	Definition:<br>Die Hauptkategorie trifft zu, wenn die IP ihre eigenen persönlichen Bedenken oder die Bedenken von anderen Personen z.B. Kolleg*innen in ihrer Einrichtung in Hinblick auf Projektinhalte erläutert, ohne das Projekt bisher umgesetzt zu haben. Die Hauptkategorie beinhaltet auch die Vorstellungen der IP über mögliche negative

**Bezeichnung der Hauptkategorie (Anzahl der Codes)**<br>▪ **Bezeichnung der Unterkategorie (Anzahl der Codes)**	**Kategoriedefinitionen, Ankerzitate & Abgrenzungshinweise**<br><br><u>Anmerkung:</u> *Jede Hauptkategorie wurde definiert. Bei den Unterkategorien wurde bis auf wenige Ausnahmen von einer Definition abgesehen. Die Referenzen für die Ankerzitate liegen der Erstautorin vor.*
	Auswirkungen in den Einrichtungen Kita und Schule mit den dort arbeitenden Personen und auf die Kinder.<br><br>Abgrenzung:<br>- Unterkategorie *Bedenken gegenüber dem Projekt* (Hauptkategorie *Persönliche Erfahrungen und Erlebnisse der IP*)<br>Beide Unterkategorien beziehen sich auf retrospektive Erfahrungswerte, die im Rahmen der Projektarbeit und der Umsetzung gesammelt wurden.<br><br>Ankerzitat:<br>*„(..) (IP räuspert sich) Ähm, wie gesagt, ich habs ja noch nicht eingesetzt. Ich KÖNnte mir ähm bei der ein oder anderen Kollegin sicherlich VORstellen, dass die dann sagen würden ähm 'Wir wollen da nichts lostreten, solange alles gut ist.' oder 'Wenn wir nicht mitbekommen, dann müssen wir nicht nachHAken oder müssen das Thema nicht aufarbeiten.'. Das könnte sicherlich nen Problem sein."*
Persönliche Erfahrungen und Erlebnisse der IP mit dem Projekt (182)	<u>Definition:</u><br>Die Hauptkategorie ist zutreffend, wenn die IP von Begebenheiten, Erlebnissen und ihren persönlichen Erfahrungen während der Projektumsetzung berichtet. Hierbei können zum Beispiel die Reaktionen der Eltern, Kinder und Mitarbeitenden in den Einrichtungen beschrieben werden. Es geht um das Erleben des Projekts durch die IP, von dem die IP retrospektiv erzählt.
▪ Bedenken gegenüber dem Projekt (49)	<u>Definition:</u><br>Sobald die IP eigene Hemmungen, Bedenken oder Sorgen äußert, die aus der Teilnahme an Projektaktivitäten hervorgehen, wird diese Unterkategorie kodiert. Auch wenn die IP äußert, welche Hemmungen und Barrieren ihr im Austausch mit anderen gegenüber der Projektumsetzung und dem Projektkonzept begegnet sind, wird diese Unterkategorie kodiert.<br><br>Ankerzitat:<br>*„Ähm. Gab eigentlich keine. Wie gesagt am Anfang vielleicht höchstens dieses 'Oh, 15 Minuten öh und heute hab ich vielleicht nur eine Stunde in der Klasse und was macht man jetzt oder so ne? Wie kriegt man das kompensiert?'."*
▪ Aufzählungen von Projekterfahrungen (11)	<u>Definition:</u><br>Die Unterkategorie ist zutreffend, wenn die IP erzählt, welche Projektaktivitäten in ihrer Einrichtung umgesetzt oder/und von ihr erlebt worden sind, ohne dabei auf einzelne inhaltliche Aspekte näher einzugehen. Auch wenn keine Erfahrungen der IP vorliegen, wird in dieser Unterkategorie kodiert.
▪ Bekanntmachung in der Einrichtung (1)	
▪ Direkte Auswirkungen durch die Projektumsetzung (45)	<u>Definition:</u><br>Die Unterkategorie trifft zu, wenn direkte Einflüsse durch die Durchführung der Projekte beobachtet oder erfahren worden sind, die in einem unmittelbaren zeitlichen Zusammenhang zu der Durchführung des Projekts stehen. Es sind sowohl Auswirkungen auf die primäre Zielgruppe der Kinder als auch auf die Projektbeteiligten beinhaltet.<br><br>Ankerzitat:<br>*„Obwohl jede Lehrkraft natürlich auch den Nutzen von diesem ganzen auch mit sieht. Und auch WELchen, wie positiv es sich auswirkt, wenn die Kinder aus dieser LAUFpause wieder rauskommen. Und wie akTIV und konzentriert sie dann halt auch weiterarbeiten können. Weil es dann insbesondere ja die VIERte Stunde, also auch zum Ende des Tages ist. Da auch nochmal son bisschen Schub gibt für die Kinder, nochmal ja klar sie sind auch kaputter in dem Moment, aber sie machen das dann eigentlich schon ganz TOLL. Man kann dann wirklich nochmal gut einsteigen dann. (...)"*
▪ Einstellungen und Reaktionen der Eltern (29)	<u>Definition</u>:<br>Die Unterkategorie trifft zu, wenn die IP über die Einstellung der Eltern gegenüber Projektinhalten spricht und Aussagen über die elterliche Einschätzung zu einem Projekt teilt.

**Bezeichnung der Hauptkategorie (Anzahl der Codes)**<br>▪ **Bezeichnung der Unterkategorie (Anzahl der Codes)**	**Kategoriedefinitionen, Ankerzitate & Abgrenzungshinweise**<br><br>Anmerkung: *Jede Hauptkategorie wurde definiert. Bei den Unterkategorien wurde bis auf wenige Ausnahmen von einer Definition abgesehen. Die Referenzen für die Ankerzitate liegen der Erstautorin vor.*
▪ Reaktionen der Kinder (47)	Definition:<br>Die Unterkategorie ist dann zutreffend, wenn die IP beschreibt, wie die Kinder auf Projektangebote und -aktivitäten reagiert haben. Sie kann dazu das Verhalten, die Gefühle und Äußerungen von Kindern schildern.
Integrierbarkeit des Projekts in der Einrichtung (134)	Definition<br>Die Hauptkategorie trifft zu, wenn die IP erzählt, ob oder wie sich das Projekt mit den Abläufen vereinbaren und in der Einrichtung (Kita oder Schule) integrieren ließ. Auch die Übereinstimmung des Konzepts eines Programmes mit der inhaltlichen Ausrichtung der Einrichtung wird unter dieser Hauptkategorie berücksichtigt.<br><br>Ankerzitat:<br>*„Also wir haben es dadurch, dass wir es in die Pausen gesetzt haben, war das möglich. So an sich zu sagen, wir nehmen jetzt 15 Minuten von einer Stunde weg fände ich etwas SCHWIErig."*
▪ Kommunikation als Voraussetzung der Integrierbarkeit (5)	Definition:<br>Wenn die IP der Kommunikation einen hohen Stellenwert für die Einführung und Durchführung eines Projektes zuspricht und die Wichtigkeit von Absprachen für das Gelingen von Projektaktivitäten hervorhebt, wird diese Unterkategorie codiert.<br><br>Abgrenzung:<br>- Hauptkategorie Zusammenarbeit von der IP mit anderen Projektbeteiligten<br>Unter dieser Haupt- und ihren Unterkategorien finden sich beschreibende Äußerungen der IP über die Art und Weise der Zusammenarbeit von Projektbeteiligten. Dabei sind auch Ausführungen der IP zur Gestaltung der Kommunikation im Team von Bedeutung.
▪ Übereinstimmung mit inhaltlicher Ausrichtung der Einrichtung (13)	Definition:<br>Die Unterkategorie wird kodiert, wenn sich aus den Beschreibungen der IP Aussagen über die Beziehung zwischen der inhaltlichen Ausrichtung der Einrichtung und den Projektinhalten ableiten lassen. Hierbei kann die IP beispielsweise auf Konflikte oder auch Schnittstellen von dem Projekt mit den Zielen und Grundsätzen der Arbeitsweise einer Einrichtung hinweisen. Außerdem kann die IP zum Beispiel auf andere Aktivitäten der Einrichtung hinweisen, die projektähnliche Ziele verfolgen.<br><br>Abgrenzung:<br>- Unterkategorie *Vereinbarkeit mit Lehrplänen und Aufträgen der Einrichtung*, (Hauptkategorie *Integrierbarkeit des Projekts in der Einrichtung*)<br>Hierbei handelt es sich um übergeordnete, festgelegte Vorgaben an die Einrichtung.
▪ Vereinbarkeit mit Lehrplänen und Aufträgen der Einrichtung (13)	Definition:<br>Die Unterkategorie wird kodiert, wenn die IP erklärt, wie sich die Projektinhalte mit Inhalten aus den Lehrplänen in Grundschulen oder mit den Kernaufgaben und -aufträgen in den Kitas vereinbaren lassen.
▪ Vereinbarkeit mit strukturellen Bedingungen (30)	Definition:<br>Die Unterkategorie wird codiert, wenn die IP darüber spricht, in welcher Beziehung sich örtlichen, zeitlichen und räumlichen Strukturen zu den Projektinhalten stehen. Dabei kann die IP zum Beispiel darüber Aussagen treffen, wie sich die Projektinhalte mit den zeitlichen Ressourcen von Einrichtungsmitarbeiter*innen vereinbaren lassen.
▪ Beschreibungen der IP zu Abänderungen von Projektinhalten (9)	Definition:<br>Unterkategorie trifft zu, wenn die IP beschreibt welche Veränderungen an der ursprünglichen Projektumsetzung vollzogen worden sind.<br><br>Ankerzitat:<br>*„Aber jetzt brauch ich ja // Wir machen das ja eigentlich auch so als wir nehmen es ja eigentlich so / Ich sag jetzt nicht The Daily. Sonst haben wir schon immer gesagt The Daily MILE und jetzt sagen wir, wie gesagt immer 'Es ist LAUFpause.'"*
▪ Auswirkung der Corona-Pandemie auf die Projektumsetzung (60)	Definition:<br>Die Hauptkategorie trifft zu, wenn die IP berichtet, inwiefern und ggf. welche pandemiebedingten Auswirkungen es auf die Projektumsetzung gegeben hat.

Bezeichnung der Hauptkategorie (Anzahl der Codes)<br>▪ **Bezeichnung der Unterkategorie (Anzahl der Codes)**	**Kategoriedefinitionen, Ankerzitate & Abgrenzungshinweise**<br><br>Anmerkung: *Jede Hauptkategorie wurde definiert. Bei den Unterkategorien wurde bis auf wenige Ausnahmen von einer Definition abgesehen. Die Referenzen für die Ankerzitate liegen der Erstautorin vor.*
	Ankerzitat:<br>*„Also ähm das Projekt war ja dann in dem Sinne ja erstmal auf Eis gelegt. Das heißt, wir haben es im GROßen Rahmen es GAR nicht mehr gemacht. Ähm wir haben dann halt geguckt, ob wir es dann wirklich KLASsenweise dann noch machen KÖNnen in einzelnen Phasen, wo dann halt wieder erlaubt war bestimmte Sachen zu machen. Ob Maske abzuziehen oder nicht, mit Maske ohne Maske. Weil wir halt gesagt haben, also mit Maske werden wir nicht LAUfen. Das möchten wir halt einfach nicht."*
▪ Einschätzungen der IP zur Integrierbarkeit in anderen Einrichtungen (4)	Definition:<br>Wenn die IP darüber spekuliert, wie das Projekt in anderen Schulen oder Kitas umgesetzt werden könnte. Unter dieser Kategorie werden auch Vorstellungen der IP über Schwierigkeiten von anderen Einrichtungen kodiert.<br><br>Abgrenzung:<br>- Hauptkategorie *Integrierbarkeit des Projekts in der Einrichtung*<br>Diese Kategorie bezieht sich nur auf die Machbarkeit und Umsetzung in der Einrichtung (Schule oder Kita), in der die IP tätig ist.
Zusammenarbeit der IP mit anderen Projektbeteiligten (139)	Definition:<br>Die Hauptkategorie trifft zu, wenn die IP erzählt, welche Unterstützungsangebote sie von anderen Projektbeteiligten in der Umsetzungsphase des Projektes erhalten hat.<br><br>Abgrenzung:<br>- Unterkategorie *Kommunikation als Voraussetzung der Integrierbarkeit* (Hauptkategorie *Integrierbarkeit des Projekts in der Einrichtung*)<br>Unter dieser Kategorie werden Aussagen über die Wichtigkeit von Kommunikation für die Durchführung von Projektaktivitäten allgemein kodiert. Hier finden sich allerdings keine Aussagen dazu, wie genau die Zusammenarbeit im Rahmen des Projekts abgelaufen ist.<br><br>Ankerzitat:<br>*„Auch der Umgang mit den KOLLEGinnen war dann da auch nochmal nen bisschen anders und es war irgendwie mehr sone Selbstverständlichkeit bei manchen Sachen da und es war jetzt klar. Das geht jetzt oder da kann ich ihr mal Hilfestellung leisten. Das war nicht ganz so son FremdgeFÜHL sag ich mal. Am Anfang ist man immer erstmal, man muss sich aneinander rantasten und so."*
▪ Wahrnehmung der IP von Zusammenarbeit (23)	Definition:<br>Wenn die IP etwas darüber aussagt, wie sie die Zusammenarbeit in Bezug auf die Projektaktivitäten wahrnimmt und erlebt. Unter dieser Kategorie finden sich auch Bewertungen der IP der Zusammenarbeit während der Projektumsetzung oder Aussagen über gewünschte Zuständigkeiten und die Übernahme von Aufgaben. Außerdem wird diese Unterkategorie kodiert, wenn die IP Voraussetzungen für eine gelingende Zusammenarbeit erläutert.<br>Auch wenn gemeinsam Bewertungen einer Intervention kodiert werden, wird diese Kategorie verwendet.<br><br>Ankerzitat:<br>*„(7) ALso ich denke ne richtige ZuSAMmenarbeit WAR es mit Fit fürs Leben, die hier her gekommen sind und hier durchgesprochen haben, was wir machen wollen / [Die waldpädagogische Fachkraft] habe ich vorher nicht gesehen. [Die] hat das gemacht, was [sie] für richtig hielt. Und das war auch GUT. Das will ich nicht in Frage stellen. ABER da gab es keine Zusammenarbeit vorher (.). Unter Zusammenarbeit verstehe ich auch was, was so fortlaufend ist. Also wo man sich nicht einmal zehn Minuten austauscht. Das ist noch keine Zusammenarbeit. Da findet man / Da weiß man einfach, wie es laufen soll ne. Mit Fit fürs Leben DENke ich WAR es gut. Also so gut, wie es durch Corona dann eben möglich war. (.)"*

Bezeichnung der Hauptkategorie (Anzahl der Codes)<br>▪ Bezeichnung der Unterkategorie (Anzahl der Codes)	Kategoriedefinitionen, Ankerzitate & Abgrenzungshinweise<br><br>Anmerkung: *Jede Hauptkategorie wurde definiert. Bei den Unterkategorien wurde bis auf wenige Ausnahmen von einer Definition abgesehen. Die Referenzen für die Ankerzitate liegen der Erstautorin vor.*
▪ Wahrnehmung der IP von Unterstützung (42)	Definition:<br>Die Unterkategorie trifft zu, wenn die IP schildert, wie sie durch Kolleg*innen oder andere Projektbeteiligte, insbesondere Ansprechpersonen aus dem Projektteam oder durch Unterstützung von Einrichtungsleitungen, Hilfestellungen bekommen hat. Darunter fällt auch das Wissen der IP um Möglichkeiten für Unterstützung und Hilfestellungen, wie beispielsweise die Kenntnis von Ansprechpersonen.<br><br>Ankerzitat:<br>*„Und vorher hatte ich halt ähm viel Unterstützung auch von den ErZIEHerinnen in den Kitas, die mir dann einfach / Also die halt am Anfang auch gar nicht wussten, was mache ich da überhaupt ne. Und als sie dann gemerkt haben ähm, was wir da ähm da brauchten / Dann haben sie sich auch selbst angeboten. 'Hier haben wir noch dies Material und das."*
▪ Wertschätzung der unterschiedlichen Expertisen (9)	Definition:<br>Diese Unterkategorie bündelt Kodiereinheiten, in denen die IP die Fähigkeiten und Expertisen von Projektbeteiligten des Projekts anerkennen. Diese Projektbeteiligten haben häufig andere Aufgaben im Rahmen des Projekts und unterscheiden sich allgemein in puncto Beruf von den IP.
▪ Kommunikation innerhalb des Projektteams (22)	Definition:<br>Die Unterkategorie trifft zu, wenn Äußerungen der IP darüber getroffen werden, wie Veränderungen und Anpassungen im bestehenden Team des laufenden Projekts oder in der Vorbereitungsphase auf das Projekt besprochen werden. Diese Kategorie wird auch kodiert, wenn die Art und Weise von Absprachen und Abstimmungen in den Erzählungen der IP deutlich werden.<br><br>*Ankerzitat:*<br>*„Ähm (.) ja und ähm die Ernährungsleute, die waren Ansprechpartner auch hinterher nach der Durchführung der Einheit haben wir auch drüber geredet. Ich weiß, wir hatten in der vierten Klasse mal ne Kakao-Einheit gemacht und die war für einige Schüler SCHWER, weil sie diese Länder, Mittelamerika und so was, das hatten sie nicht so parat. Und ähm (.) da haben wir gesagt 'Da müssen wir da nochmal nen bisschen aufARbeiten hinterher.'. Also das wurde schon transparent gemacht und da konnten wir drüber reden. JA. (.)"*
▪ Erleben der Beteiligung an Projektentscheidungen (28)	Definition:<br>Die Unterkategorie trifft zu, wenn die IP erzählt, wie sie Entscheidungen im Projektverlauf trifft oder wie sie in Entscheidungen eingebunden worden ist.
▪ Bereitstellung von Sachmaterialien für die IP (15)	Definition:<br>Die Unterkategorie trifft zu, wenn die IP erzählt, welche Materialien sie von anderen Projektbeteiligten für die Umsetzung des Projekts erhalten hat. Sie wird auch kodiert, wenn die IP erwähnt, dass ihr keine Materialien zur Verfügung standen.<br><br>Ankerzitat:<br>*„Und hab auch geguckt, ob es dazu irgendwelches MateriAL schon gibt oder ne es gibt ja häufig so Materialien. Ist nicht so, dass mir die häufig GUT geFALlen, aber manchmal HELfen die mir. Dass ich für MICH, dass ich mir da Sachen rausziehe. Und das so verändere, dass es für meine Gruppe dann irgendwie PASST. Ähm ich hab allerdings NICHTS gefunden dazu. (IP lacht) (.) Joa. (…)"*
Sichtweisen der IP zu langfristigen Wirkungen des Projekts (35)	Definition:<br>Die Hauptkategorie trifft zu, wenn die IP einschätzen, ob und inwiefern sich die Projekte auch über das Projektende hinaus auf die Gesundheit der Kinder auswirken könnten.<br><br>Ankerzitat:<br>*„Joa also ich denke schon, dass viele Kinder auch durch sowas wie das Mini-Sportabzeichen irgendwie mal (.) Selbstvertrauen kriegen und irgendwie Sachen haben, wo sie stolz drauf sein können, dass sie das geschafft haben ähm mit ihren einzelnen kleinen Defiziten vielleicht irgendwie das TROTZdem hingekriegt haben diesen Parcour zu machen oder irgendwie ähm (überlegend) (.) ja das und, dass da einfach auch so Interessen entwickelt werden."*

**Bezeichnung der Hauptkategorie (Anzahl der Codes)**<br>▪ **Bezeichnung der Unterkategorie (Anzahl der Codes)**	**Kategoriedefinitionen, Ankerzitate & Abgrenzungshinweise**<br><br>Anmerkung: *Jede Hauptkategorie wurde definiert. Bei den Unterkategorien wurde bis auf wenige Ausnahmen von einer Definition abgesehen. Die Referenzen für die Ankerzitate liegen der Erstautorin vor.*
▪ Auswirkungen in der Einrichtung (5)	Definition:<br>Wenn die IP erzählt, wie dauerhaft Themen oder Inhalte aus Projekten oder Programmen innerhalb der Umsetzung durch Mitarbeitende selbstständig umgesetzt werden könnten, trifft diese Unterkategorie zu.
▪ Auswirkungen in der Zielgruppe (26)	Definition:<br>Die Hauptkategorie wird kodiert, wenn die IP Auswirkungen in der Zielgruppe wahrnimmt, die mit der Durchführung einer Intervention in Verbindung stehen. Dabei handelt es sich um Auswirkungen, die über die Durchführung der Intervention andauern können; wie z. B., dass Kinder außerhalb des Projekts Bewegungsangebote wahrnehmen oder Ressourcen durch das Projekt aufbauen konnten, die sich fortwährend auf die Entwicklung auswirken könnten.
▪ Wahrnehmung der IP von Projektwirkungen (4)	Definition:<br>In dieser Unterkategorie werden Zweifel und Unsicherheiten der IP kodiert. Wenn die IP Zweifel daran hat, ob langfristige Wirkungen vorhanden sind oder keine Aussagen aus Sicht der IP darüber möglich sind, wird diese Kategorie kodiert. Die Kategorie wird auch kodiert, wenn die IP keine langfristigen Wirkungen durch die Projektumsetzung festmachen kann.
Gedanken der IP zu unmittelbaren Auswirkungen des Projektes (10)	Definition:<br>Die Hauptkategorie trifft zu, wenn die IP ihre Vorstellungen darüber preisgibt, wie sich die Durchführung von Projektinhalten auf die Zielgruppe unmittelbar, zeitnah auswirken könnte. Diese Vorstellungen basieren auf den von der IP erwarteten Wirkungen, ohne dass es bisher zu der Durchführung und Anwendung von Projektinhalten gekommen ist.<br><br>Hinweis: Diese Kategorie findet nur für Aussagen zu der Intervention *Sonnige Traurigtage Anwendung*.<br><br>Abgrenzung:<br>- Unterkategorie *Direkte Auswirkungen durch die Projektumsetzung* (Hauptkategorie *Persönliche Erfahrungen und Erlebnisse der IP*)<br>In dieser Hauptkategorie bilden Erfahrungen die Basis für die Aussagen der IP. Sie berichtet retrospektiv über Erlebnisse.<br><br>Ankerzitat:<br>*„(..) SCHWER zu sagen. Ähm (.) weils SO viele unterschiedliche Kinder in unterschiedlichen KLASsen mit unterSCHIEDlichen ÄHM häuslichen Situationen gibt. Ähm dass eigentlich alles passieren könnte. (.). Das kann positiv sein, das kann negativ sein. (..) Und auch alles dazwischen. (.)“*
▪ antizipierte positive Wirkungen (3)	Definition:<br>Die Unterkategorie wird kodiert, wenn die IP von der Projektdurchführung positive Wirkungen erwartet.
▪ antizipierte negative Wirkungen (3)	Definition:<br>Die Unterkategorie wird kodiert, wenn die IP von der Projektdurchführung negative Wirkungen erwartet.
▪ keine eindeutige Bewertung (4)	Definition:<br>Die Unterkategorie wird verwendet, wenn die IP sowohl positive als auch negative Auswirkungen erwartet und sich nicht klar positioniert bei der Bewertung möglicher unmittelbarer Auswirkungen.
Meinung der IP zum Fortgang des Projekts (48)	Definition:<br>Die Hauptkategorie trifft zu, wenn die IP ihre Vorstellungen, Ideen und Fantasien, Mutmaßungen über die Projektzukunft beschreiben.<br>Auch Wünsche für die Art der Fortsetzung des Projekts sowie zur Projektgestaltungen sind hierunter zu verstehen.<br><br>Ankerzitat:<br>*„Also ich hoffe einfach, dass ich mit dem Buch wirklich mal richtig ansetzen kann und arbeiten KANN. Und ähm (.) muss natürlich ne Bereitschaft auch da sein von ähm (.) oder vielleicht mehr einer Erlaubnis VON Elternseite. Und ähm dann würde ich das gerne mitaufgreifen und ähm (..)“*

**Bezeichnung der Hauptkategorie (Anzahl der Codes)**<br>▪ **Bezeichnung der Unterkategorie (Anzahl der Codes)**	**Kategoriedefinitionen, Ankerzitate & Abgrenzungshinweise**<br><br>*Anmerkung: Jede Hauptkategorie wurde definiert. Bei den Unterkategorien wurde bis auf wenige Ausnahmen von einer Definition abgesehen. Die Referenzen für die Ankerzitate liegen der Erstautorin vor.*
▪ Ausprägung der zukünftigen Umsetzung (35)	
▪ Weiterentwicklung der Projektgestaltung (13)	
Sonstiges (13)	Definition:<br>Die Hauptkategorie trifft zu, wenn keine der anderen Hauptkategorien zutreffend ist.
